# Efficacy of Different Interventions to Reduce Pre- or Perioperative Blood Transfusion Rate in Patients with Colorectal Cancer: A Network Meta-Analysis of Randomized Controlled Trials

**DOI:** 10.3390/curroncol28040279

**Published:** 2021-08-22

**Authors:** Chao-Ming Hung, Jiann-Jy Chen, Bing-Yan Zeng, Bing-Syuan Zeng, Yen-Wen Chen, Mein-Woei Suen, Ming-Kung Wu, Ping-Tao Tseng

**Affiliations:** 1 Division of General Surgery, Department of Surgery, E-Da Cancer Hospital, Kaohsiung 82445, Taiwan; ed100647@edah.org.tw; 2 School of Medicine, College of Medicine, I-Shou University, Kaohsiung 84001, Taiwan; 3 Department of Otorhinolaryngology, E-Da Cancer Hospital, Kaohsiung 82445, Taiwan; jiannjy@yahoo.com.tw; 4 Prospect Clinic for Otorhinolaryngology & Neurology, Kaohsiung 811, Taiwan; kevinachen0527@gmail.com; 5 Department of Internal Medicine, E-Da Hospital, Kaohsiung 82445, Taiwan; holdinggreat@yahoo.com.tw (B.-Y.Z.); b95401072@ntu.edu.tw (B.-S.Z.); 6 Department of Psychology, College of Medical and Health Science, Asia University, Taichung 413, Taiwan; blake@asia.edu.tw; 7 Gender Equality Education and Research Center, Asia University, Taichung 413, Taiwan; 8 Department of Medical Research, Asia University Hospital, Asia University, Taichung 413, Taiwan; 9 Department of Medical Research, China Medical University Hospital, China Medical University, Taichung 406040, Taiwan; 10 Department of Psychiatry, Kaohsiung Chang Gung Memorial Hospital, Chang Gung University College of Medicine, Kaohsiung 83301, Taiwan; 11 Institute of Biomedical Sciences, National Sun Yat-Sen University, Kaohsiung 80424, Taiwan

**Keywords:** colorectal cancer, anemia, erythropoietin, iron, network meta-analysis

## Abstract

Background: The high proportion of blood transfusions before and during surgery carries unnecessary risk and results in poor prognosis in colorectal cancer patients. Different pharmacological interventions (i.e., iron supplement or recombinant erythropoietin) to reduce blood transfusion rates have shown inconclusive results. Methods: This network meta-analysis (NMA) consisted of randomized controlled trials (RCTs) comparing the efficacy of different pharmacologic interventions (i.e., iron supplementation or recombinant erythropoietin) to reduce the blood transfusion rate. NMA statistics were conducted using the frequentist model. *Results:* Seven RCTs (688 participants) were included in this study. The NMA demonstrated that the combination of high-dose recombinant human erythropoietin and oral iron supplements was associated with the least probability of receiving a blood transfusion [odds ratio = 0.24, 95% confidence intervals (95% CIs): 0.08 to 0.73] and best reduced the amount of blood transfused if blood transfusion was necessary (mean difference = −2.62 U, 95% CI: −3.55 to −1.70 U) when compared to the placebo/control group. None of the investigated interventions were associated with any significantly different dropout rate compared to the placebo/control group. Conclusions: The combination of high-dose recombinant human erythropoietin and oral iron supplements might be considered as a choice for reducing the rate of blood transfusion in patients with colorectal cancer. However, future large-scale RCT with long-term follow-up should be warranted to approve the long-term safety.

## 1. Introduction

According to the GLOBOCAN 2018 [1], colorectal cancer is the fourth most common cancer in incidence and the second leading cause of cancer death in both sexes. Early surgical intervention in resectable colorectal cancer has become a widely accepted choice for the management of colorectal cancer. However, several pre-operative risk factors predominantly affect the prognosis of surgery. Anemia, a frequent comorbidity of colorectal cancer [2], is a pre-operative risk factor that modifies the prognosis of surgery [3]. In addition to the adverse impact of anemia, the necessity to receive allogeneic blood transfusion because of its severity has brought extra potential risks. Specifically, allogeneic blood transfusion has been found to be associated with increased risks for immunosuppression [4], disease transmission, and allergic reaction. In addition, two previous meta-analyses found that allogeneic blood transfusion may be associated with an increased risk for colorectal cancer recurrence; however, results remained inconclusive [5,6]. To resolve the potential risks of allogeneic blood transfusion, autotransfusion has become an alternative method. However, this method also brought an additional risk for reintroducing tumor cells into the patient [7]. Furthermore, only a few colorectal cancer patients were eligible for autotransfusion; therefore, their usage was highly limited [8,9]. However, because of complications such as anemia, the need for blood transfusion during surgery in colorectal cancer patients still exists and is as high as 45–80% [7]. Therefore, reducing the need for pre-operative or perioperative blood transfusions has become an unavoidable issue in colorectal cancer surgery.

Several predictors of increased pre-operative or perioperative blood transfusion have been found, including large tumor size, operative blood loss, and pre-operative anemia [10]. Among these predictors, only pre-operative anemia can be modified before the surgical procedure. Therefore, several interventions to modify pre-operative anemia have been developed, such as oral and intravenous iron supplements and as well as recombinant human erythropoietin. Although some clinical trials have shown a significantly beneficial effect on the pre-operative anemia profile with iron therapy [10,11], the recent meta-analyses of iron therapy for pre-operative anemia did not provide a statistically significant result [12,13]. Similarly, although the administration of recombinant human erythropoietin could theoretically stimulate erythropoiesis in a dose-dependent manner [14] and relieve the need for blood transfusion, there has been limited evidence regarding the ability of recombinant human erythropoietin to reduce the need for blood transfusion in patients undergoing surgery for bowel/colorectal cancer. In one recent Cochrane meta-analysis by Devon and McLeod, which merged different dosage of erythropoietin into one group, there was no statistically significantly different transfusion rate between the erythropoietin group and control group [15]. In addition, there were only some placebo-controlled randomized controlled trials (RCTs) to investigate the efficacy of recombinant human erythropoietin in anemia profiles; none of these RCTs provided extra information about the multiple comparisons between different active treatments [7,16,17,18]. Therefore, knowledge about the superiority of each intervention is limited.

A network meta-analysis (NMA) enables comparative estimation of the efficacy of multiple interventions and understanding of the relative merits of each one. Moreover, an NMA provides additional information on clinical practice reference, which cannot be achieved by traditional pairwise meta-analyses [19]. To the best of our knowledge, to date, no NMA has compared the efficacy of different pharmacological interventions, (i.e., iron supplement or recombinant erythropoietin) to reduce blood transfusion rate. Therefore, we aimed to conduct a systematic review and NMA by comparing different pharmacologic interventions (i.e., iron supplement or recombinant erythropoietin) to reduce the blood transfusion rate.

## 2. Materials and Methods

### 2.1. General Guidelines Applied in This Study

This NMA followed the guidelines for preferred reporting items for systematic reviews and meta-analyses (PRISMA) 2020 [20] (Table S1) and assessing the methodological quality of systematic reviews (AMSTAR2) [21]. The overall structure and flowchart of the current NMA were designed according to a previous important meta-analysis or NMAs [22,23,24,25,26].

### 2.2. Search Strategy and Selection Criteria

We conducted a systematic review of the following databases: PubMed, ClinicalKey, Cochrane CENTRAL, Embase, ProQuest, ScienceDirect, and Web of Science from inception to 25 April 2021. We used the following keywords: (iron OR ferritin OR ferric OR ferrous OR erythropoietin OR epoetin alfa OR EPO OR hemopoietin OR haemopoietic OR epoetin beta OR hematopoietin OR erythrogenin OR erythrogenic OR erythropoietin) AND (colorectal cancer OR colorectal neoplasm OR colorectal carcinoma OR colorectal tumor OR colon cancer OR colon neoplasm OR rectal cancer OR rectum cancer OR rectal neoplasm OR rectum neoplasm OR colon tumor OR rectum tumor OR rectal tumor) AND (random OR randomized OR randomized) (detailed search strategy was depicted in Table S2). In order to find unpublished studies or gray literature, we also electronically searched ClinicalTrials.gov. No language restrictions were imposed. We also conducted manual searches for potentially eligible articles from the reference lists of review articles and pairwise meta-analyses [13,15,27].

### 2.3. Inclusion and Exclusion Criteria

The PICO of the current study was as follows: (1) patient or problem: colorectal patients who planned to undergo bowel/colorectal surgery; (2) intervention: pharmacologic intervention, (i.e., iron supplement or recombinant erythropoietin) to reduce the blood transfusion rate; (3) comparator: placebo-controlled, waiting-list, or active-controlled, and (4) outcome: the blood transfusion rate during surgery.

We only included RCTs with either placebo-controlled, waiting-list, or active-controlled design that were conducted in adult humans with colorectal cancer who planned to undergo surgery. The inclusion criteria were as follows: (1) human RCTs, (2) clinical trials recruiting patients with colorectal cancer who were scheduled to undergo surgery, and (3) trials with pre-operative intervention, (i.e., iron supplement or recombinant erythropoietin) to reduce the blood transfusion rate.

The exclusion criteria were as follows: (1) non-RCTs, (2) a protocol, but not a report of the study result, (3) trials recruiting patients with cancers other than colorectal cancer, or (4) trials not associated with pre-operation intervention (i.e., iron supplement or recombinant erythropoietin) to reduce the blood transfusion rate. In cases of data duplication (i.e., different articles based on the same sample sources), we only included the study that was the most informative and had the largest sample size.

### 2.4. Data Extraction

Two authors (MK Wu and PT Tseng) independently screened the studies, extracted the relevant data from the manuscripts, and assessed the risk of bias among the included studies. In situations of discrepancy, the third author was consulted for final decision making. If the eligible data were lacking in the included manuscripts, we contacted the corresponding authors or co-authors to obtain the same. If one study provided pre-operative, perioperative, and postoperative intervention, we only extracted the outcome data just before the target surgery (i.e., bowel/colorectal surgery).

### 2.5. Primary Outcome

The primary outcome was the rate of blood transfusion during the target surgery (i.e., bowel/colorectal surgery).

### 2.6. Secondary Outcomes

The secondary outcomes were the changes in hemoglobin level (converted into uniform units of g/dL), ferritin level (converted into uniform units of ng/mL), and the amount of blood transfused (calculated in uniform units of “Units”).

### 2.7. Acceptability

Treatment acceptability was evaluated by assessing the dropout rate, which was defined as participants leaving the trials before reaching completion for any reason. We chose this definition according to the rationale of a previously published NMA in *Lancet Psychiatry* [28], in which the authors defined their acceptability as a dropout rate.

### 2.8. Cochrane Risk of Bias Tool and Quality of Evidence Evaluation

Two independent authors evaluated the risk of bias (inter-rater reliability, 0.88) for each domain, as described in the Cochrane risk of bias tool [29].

### 2.9. Statistical Analysis

The NMA was performed using Stata software (version 16.0; StataCorp LLC Statistics/Data Analysis StataCorp, College Station, TX, USA). For continuous data, we estimated the summary mean difference (MD) with 95% confidence intervals (95% CIs). For categorical data, we estimated the summary odds ratio (OR) with 95% CIs. Additionally, for categorical data, we applied a 0.5 zero-cell correction during the meta-analysis. However, if there were zeroes in both the intervention and control arms, such a correction procedure was not applied because of the risk of increasing the bias [30,31]. We used frequentist models of the NMA to compare the different interventions. Regarding the meta-analysis conducted in this study, we used a mixed treatment comparison with a generalized linear mixed model to analyze the indirect and direct comparisons in the NMA [32]. To compare the multiple treatment arms, we combined direct and indirect evidence from the included studies [33]. The *mvmeta* package of the Stata program was used in our NMA [32]. When only “median + interquartile range” or “median + upper/lower limit” data were available, we transformed those data into “mean + standard deviation” according to the statistical rationale raised in previous publications [34,35]. Finally, to reduce the potential heterogeneity, we performed a subgroup analysis according to RCTs recruiting patients with baseline anemia.

Furthermore, we calculated the surface under the cumulative ranking curve (SUCRA), which is the percentage of the mean rank of each intervention, to rank the relative superiority of the investigated intervention [36]. We used the comparison-adjusted funnel plot and Egger regression to evaluate potentially small study effects in the order of individual treatment efficacy [37]. Finally, the potential inconsistency between the direct and indirect evidence within the network was evaluated using the node-splitting method [38]. Finally, we evaluated the quality of evidence using the Grading of Recommendations Assessment, Development, and Evaluation (GRADE) rating tools described previously [25,39].

## 3. Results

**Figure 1:**
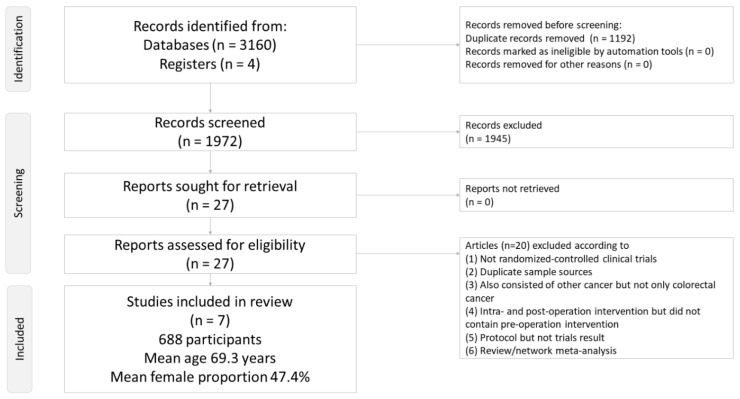
Flowchart of the current network meta-analysis.

**Figure 2:**
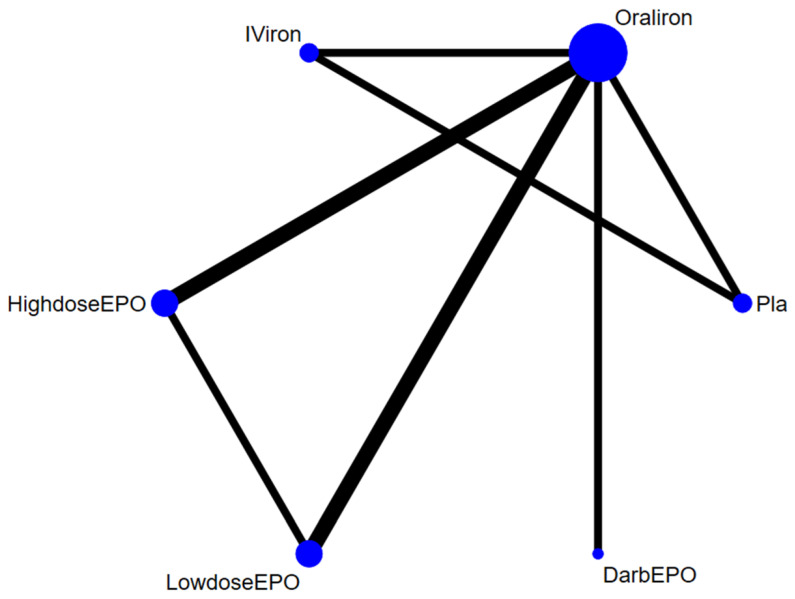
Network structure of the current network meta-analysis of primary outcome: rate of blood transfusion. Figure 2 shows the structure of the network meta-analysis for primary outcome: rate of blood transfusion; the lines between nodes represent direct comparisons in various trials, and the size of each circle is proportional to the size of the population involved in each specific treatment. The thickness of the lines is proportional to the number of trials connected to the network.

Following the initial screening procedure, a total of 27 articles were considered for full-text review (Figure 1). However, 20 of these were excluded for various reasons (Figure 1 and Table S3). In addition, the studies conducted by Dickson [2] and Keeler [40] were duplicated with another RCT, which was included in the current NMA [41]. Similarly, the study by Qvist [42] was duplicated with another RCT, which was also included in the current NMA [18]. In the study by Kettelhack [43], the intervention was administered during the intra- and post-operation periods, but not during the pre-operation period. Finally, a total of seven articles were included in this study (Table S4) [7,10,11,16,17,18,41]. Figure 2 shows the complete geometric distribution of the treatment arms. In the studies by Qvist [18], Heiss [16], Christodoulakis [7], and Norager [17], the authors administered their intervention during the pre-operation, intra-operation, and post-operation periods, thereby allowing us to extract the outcome data of the pre-operation period (i.e., the outcome data just before surgery).

### 3.1. Characteristics of the Included Studies

Among these seven RCTs, a total of 688 participants were included at baseline (mean age = 69.3 years, range: 64.0–74.3 years; mean female proportion = 47.4%, range: 34.7–66.7%; mean treatment duration = 10.6 days, range: 1–21 days). The protocol for blood transfusion was as follows: (1) hemoglobin (Hb) 8–10 cut-off points (Hb < 8 g/dL: transfusion; Hb 8-10: transfuse in specific situations) [10,11]; (2) transfusion should be considered when Hb < 8 g/dL, and should be indicated when Hb < 7 g/dL [41]; (3) transfusion should be recommended when Hb < 9 g/dL [7,16], and (4) determined by the attending anesthesiologist and surgeon according to the clinical condition of each patient [17,18]. Therefore, almost all included RCTs administered their blood transfusion according to an objective criterion, except for two RCTs, which were both well-designed double-blind studies [17,18]. Additionally, among all included RCTs, two did not set a specific Hb level as their inclusion/exclusion criteria [10,11], whereas the other five had specifically selected patients with anemia as their inclusion criteria [7,16,17,18,41].

### 3.2. Primary Outcome: Rate of Blood Transfusion

**Figure 3:**
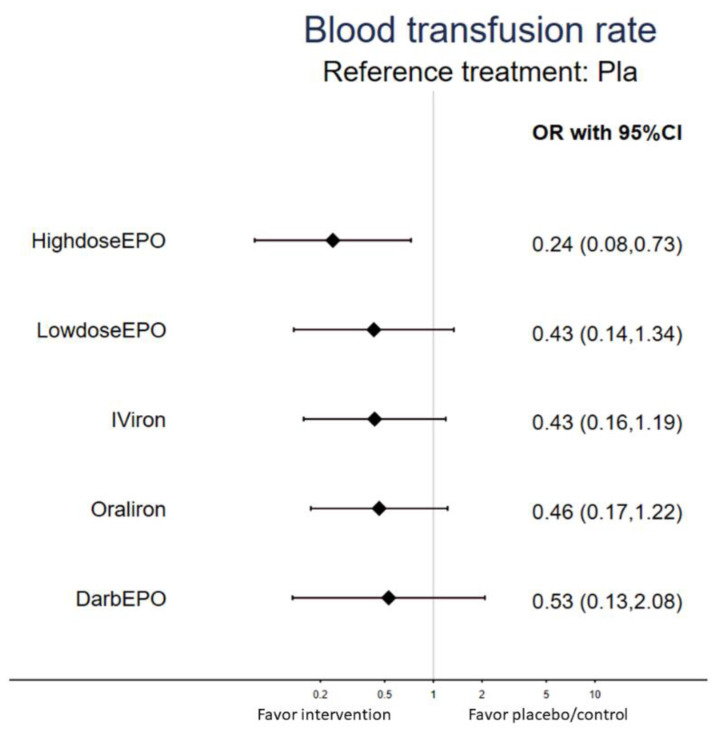
Forest plot of the current network meta-analysis of primary outcome: rate of blood transfusion. Figure 3 demonstrates that an OR < 1 indicates less rate of blood transfusion by the specified interventions than the placebo/control group.

The NMA revealed that a high dosage of recombinant human erythropoietin (epoetin alfa) 300 IU/kg *plus* oral iron supplements (200 mg/day) (HighdoseEPO) was associated with a significantly lower rate of blood transfusion than the placebo/control group (OR = 0.24, 95% CI: 0.08 to 0.73) (Table 1 and Figure 3). On SUCRA analysis, results revealed that HighdoseEPO provided the lowest rate of blood transfusion among all the other treatment interventions (Table S5A).

The main findings of the current NMA remained similar in the subgroup of RCTs recruiting patients with baseline anemia. Specifically, HighdoseEPO was ranked to provide the lowest rate of blood transfusion among all the other treatment interventions according to SUCRA (Table S5B). In addition, HighdoseEPO was associated with a significantly lower rate of blood transfusion than did the oral iron supplement only group (OR = 0.52, 95% CI: 0.30 to 0.89) (Table S6A, Figures S1A and S2A).

### 3.3. Secondary Outcome: Changes in Hemoglobin Level

The NMA revealed that none of the investigated interventions were associated with any significant difference in Hb levels compared to the placebo/control group (Tables S5C and S6B, Figures S1B and S2B).

### 3.4. Secondary Outcome: Changes in Ferritin Level

The NMA revealed that none of the investigated interventions were associated with any significant difference in ferritin levels compared to the placebo/control group (Tables S5D and S6C, Figures S1C and S2C).

### 3.5. Secondary Outcome: Changes in the Amount of Blood Transfused

The NMA revealed that all of the investigated interventions were associated with significantly lower amounts of transfused blood than the placebo/control group (Tables S5E and S6D, Figures S1D and S2D). On SUCRA analysis, HighdoseEPO was ranked to be associated with the least amount of blood transfused among all the other treatment interventions (MD = −2.62 U, 95%CIs: −3.55 to −1.70 U) (Table S6D).

### 3.6. Association between Individual Interventions and Acceptability with Respect to Dropout Rates

The results showed that none of the investigated interventions were associated with any significantly different drop-out rate compared to the placebo/control group (Tables S5F and S6E, Figures S1E and S2E).

### 3.7. Risk of Bias, Publication Bias, Inconsistency, and GRADE Ratings

We found that 63.3% (31 of 49 items), 24.5% (12 of 49 items), and 12.2% (6 of 49 items) studies had an overall low, unclear, and high risk of bias, respectively. Additionally, the occurrence of an unclear or high risk of bias was mainly distributed in the items of “concealment,” ”blind to participants,” and “blind to investigators” (Figure S3A,B). A funnel plot for assessing publication biases revealed a generally symmetrical distribution. Additionally, the results of the Egger’s test indicated no significant publication bias among the articles included in our NMA (Figure S4A–L). Detailed information on the evaluation of network inconsistency and estimated between-studies standard deviation are shown in Tables S7 and S8, respectively. In general, the results did not demonstrate local inconsistency as assessed using the loop-specific or node-splitting method, or that of global inconsistency as assessed using the design-by-treatment method. The results of the GRADE evaluation are listed in Table S9.

## 4. Discussion

To the best of our knowledge, this is the first NMA to compare the efficacy of different pharmacological interventions (i.e., iron supplements or recombinant erythropoietin) to reduce the blood transfusion rate. The main findings of our NMA revealed that the high-dose EPO (i.e., high dosage of recombinant human erythropoietin (epoetin alfa) 300 IU/kg plus oral iron supplements (200 mg/day) was ranked to be associated with the lowest rate of blood transfusion and the least amount of blood transfused among all the other treatment interventions. This finding remained similar when we focused on patients with baseline anemia. Finally, none of the investigated interventions were associated with any significantly different acceptability (i.e., drop-out rate) compared to the placebo/control group.

The most important finding of the current NMA was that the combination of high-dose recombinant human erythropoietin and oral iron supplements was associated with the least probability of receiving blood transfusion and was the best in terms of reducing the amount of blood transfused if blood transfusion was necessary. Among the included RCTs, there were two with this treatment arm (i.e., high dosage of recombinant human erythropoietin (epoetin alfa) 300 IU/kg plus oral iron supplements (200 mg/day) [7,18]. Both RCTs recruited patients with colorectal cancer and with baseline anemia. In patients with colorectal cancer, the etiology of anemia could be multifactorial, including chronic blood loss [7], iron-deficiency [44], and suppressed hematopoiesis [16]. Among them, iron-deficiency anemia accounted for the most frequent etiology, with a high prevalence of 51.9% [44]. Therefore, the prescription of oral iron supplements would be a good rationale for dealing with anemia associated with colorectal cancer. However, although oral iron supplementation alone may be able to correct iron deficiency it may not be capable of stimulating erythropoiesis to a sufficient degree in patients with gastrointestinal cancer [45]. Therefore, the additional high dosage of recombinant human erythropoietin could provide benefits in two ways. First, in iron-deficient anemia, a high dosage of recombinant human erythropoietin could boost erythropoiesis to a sufficient degree in a dose-dependent manner [14]. Second, in patients without iron-deficient anemia, the high dosage of recombinant human erythropoietin could contribute to significantly higher pre-operative Hb concentrations than in patients with iron deficiency [16]. Therefore, the combination of high-dose recombinant human erythropoietin and oral iron supplements might be a potential choice to manage anemia in patients with colorectal cancer. However, although there was no significantly different drop-out rate noted between high-dose recombinant human erythropoietin *plus* oral iron supplement group and the placebo/control groups, the safety of high-dose recombinant human erythropoietin in patients with colorectal cancer should be cautious. In the RCT by Qvist et al. [18], one patient in the treatment group developed deep venous thrombosis, although in another study there was no evidence that the perioperative treatment with high-dose recombinant human erythropoietin would influent the hemostatic parameters [46]. In the RCT by Christodoulakis et al. [7], there was only one study drug-related adverse event (i.e., incidence of grade two rash) noted, which was consistent with the findings in the review article of safety of erythropoietin [47]. Nevertheless, there was one major concern about the risk of potential enhancement of tumor growth by high-dose recombinant human erythropoietin in cancer patients. To be specific, in the mice study by Rupertus et al. [48], administration of darbepoetin-alpha alone would slightly affect the tumor metastatic growth; however, in mice receiving both darbepoetin-alpha administration and partial hepatectomy, the colorectal liver metastatic growth would be enhanced significantly. Similarly, in another RCT of head/neck cancer patients receiving radiotherapy [49], loco-regional progression-free survival was poorer in patients receiving epoetin beta than those with placebo. Although these phenomena had not been seen in the included RCTs [7,10,11,16,17,18,41], the potential risk of potential enhancement of tumor growth by high-dose recombinant human erythropoietin still could not be excluded because the risk of potential enhancement of tumor growth need longer follow-up duration to approve its existence. The clinicians needed to be cautious about the potential risk of enhancement of tumor growth when applying the high-dose recombinant human erythropoietin in cancer patients. Therefore, future large-scale RCT with long-term follow-up duration should be warranted to approve the long-term safety of high-dose recombinant human erythropoietin in cancer patients.

Another issue to be addressed is the alteration in Hb concentrations. Specifically, although the combination of high dosage recombinant human erythropoietin and oral iron supplements could relatively improve the Hb concentrations with 1.50 g/dL (95% CI: −1.58 to 4.58) compared to the placebo/control group, this difference was not statistically significant. If we consider the significant finding in the blood transfusion rate, one hypothesis could be proposed: the combination of high-dose recombinant human erythropoietin and oral iron supplements may not only target the Hb levels, but also reduce anemia-related symptoms. In most of the included RCTs, the protocol to provide blood transfusion consisted of one item: “transfuse blood if patients has clinical discomfort”; that is, the clinicians administered blood transfusion in consideration of not only absolute Hb levels, but also the patients’ clinical symptoms. The administration of recombinant human erythropoietin has been proved to reduce acute lung injury and multiple organ failure/dysfunction in a rat model [50]. Similarly, the administration of recombinant human erythropoietin also had a renal protective effect in pre-dialysis patients [51]. Furthermore, the administration of recombinant human erythropoietin could also improve the quality of life of cancer patients receiving chemotherapy [52]. Therefore, the administration of recombinant human erythropoietin may not only passively improve patients’ hematologic profiles, but also aggressively improve and preserve their quality of life and organ function. However, since none of the included RCTs provided data on the detailed reason for blood transfusion in each patient, we could not make any further analysis to clarify the underlying mechanism as to how the combination of high dosage recombinant human erythropoietin and oral iron supplements could significantly reduce the blood transfusion rate, but not significantly improve the Hb concentrations.

### Limitations

Although we tried to enhance the strength of the current meta-analysis with strict inclusion criteria (i.e., only included RCTs, only included colorectal cancer), several limitations of our study should be considered when interpreting the results. First, our NMA could be underpowered because of sample heterogeneity (e.g., colorectal cancer stage, definition of baseline anemia, protocol of blood transfusion, age and sex distribution, and trial duration). Second, the overall number of included RCTs and patients was relatively small (only seven RCTs and 688 patients). Third, although there was no severe adverse event associated with the treatment in the current meta-analysis, the concerns about the risk of potential enhancement of tumor growth by high-dose recombinant human erythropoietin in cancer patients still should be kept in mind. Since there is no clear and conclusive evident association between the worsening prognosis and transfusion only, the high-dose recombinant human erythropoietin and iron supplementation in cancer patients still should be preserved to those without other alternative choices. Finally, some of the included RCTs administered blood transfusion by the attending anesthesiologist and surgeon in accordance with the clinical condition of each patient and not according to objective criteria [17,18]. Although the risk of potential bias in selecting the patients for blood transfusion was reduced by the well-designed double-blind process in both RCTs, it may still contribute to a potential confounding bias.

## 5. Conclusions

Our NMA showed that the combination of high-dose recombinant human erythropoietin and oral iron supplements was associated with the least probability of receiving blood transfusion and was the best in terms of reducing the amount of blood transfused if blood transfusion was necessary. However, because of the limitations of this study, the overall evidence was not sufficiently strong. Future larger-scale, long-term follow-up, well-designed double-blind RCTs, with objective criteria to administer blood transfusion, are warranted to support or refute the present study results and to approve the long-term safety of high-dose recombinant human erythropoietin in cancer patients.

## Figures and Tables

**Table 1:** League table of the blood transfusion rate.

HighdoseEPO	0.61 (0.31, 1.22)		* 0.50 (0.29, 0.86)		
0.56 (0.30, 1.05)	LowdoseEPO		0.96 (0.52, 1.77)		
0.55 (0.21, 1.46)	0.99 (0.36, 2.70)	IViron	0.75 (0.30, 1.85)		0.72 (0.19, 2.82)
* 0.52 (0.30, 0.89)	0.93 (0.51, 1.68)	0.94 (0.42, 2.11)	Oraliron	0.87 (0.33, 2.29)	0.31 (0.09, 1.03)
0.45 (0.15, 1.36)	0.81 (0.26, 2.51)	0.82 (0.23, 2.89)	0.87 (0.33, 2.29)	DarbEPO	
* 0.24 (0.08, 0.73)	0.43 (0.14, 1.34)	0.43 (0.16, 1.19)	0.46 (0.17, 1.22)	0.53 (0.13, 2.08)	Pla

Pairwise (upper-right portion) and network (lower-left portion) meta-analysis results are presented as estimate effect sizes for the outcome of blood transfusion rate in patients with colorectal cancer. Interventions are reported in order of mean ranking of efficacy; outcomes are expressed as odds ratio (OR) (95% confidence intervals). For the pairwise meta-analyses, an OR < 1 indicates that the treatment specified in the row had better efficacy (i.e., a lower blood transfusion rate) than that specified in the column. For the network meta-analysis (NMA), an OR < 1 indicates that the treatment specified in the column had better efficacy (i.e., a lower blood transfusion rate) than that specified in the row. Bold results marked with * indicate statistical significance. HighdoseEPO: epoetin alfa 300 IU/kg; LowdoseEPO: epoetin alfa 150 IU/kg; IViron: intravenous iron supplement; Oraliron: oral iron supplement; DarbEPO: darbepoetin alfa; Pla: placebo/Control.

## Data Availability

All the data were available upon reasonable request.

## Supplementary Materials

The following are available online at https://www.mdpi.com/article/10.3390/curroncol28040279/s1. Figure S1: (A) network structure of NMA of rate of blood transfusion: subgroup of baseline anemia; (B) network structure of NMA of changes of hemoglobin; (C) network structure of NMA of changes of ferritin; (D) network structure of NMA of amounts of blood transfused; (E) network structure of NMA of acceptability: in aspect of drop-out rate, Figure S2: (A) forest plot of NMA of rate of blood transfusion: subgroup of baseline anemia; (B) forest plot of NMA of changes of hemoglobin; (C) forest plot of NMA of changes of ferritin; (D) forest plot of NMA of amounts of blood transfused; (E) forest plot of NMA of acceptability: in aspect of drop-out rate, Figure S3: (A) overview of risk of bias; (B) detailed risk of bias in each study, Figure S4: (A) Funnel plot of primary outcome: rate of blood transfusion; (B) Egger’s regression of primary outcome: rate of blood transfusion; (C) Funnel plot of rate of blood transfusion: subgroup of baseline anemia; (D) Egger’s regression of rate of blood transfusion: subgroup of baseline anemia; (E) Funnel plot of changes of hemoglobin; (F) Egger’s regression of changes of hemoglobin; (G) Funnel plot of changes of ferritin; (H) Egger’s regression of changes of ferritin; (I) Funnel plot of amounts of blood transfused; (J) Egger’s regression of amounts of blood transfused; (K) Funnel plot of acceptability: in aspect of drop-out rate; (L) Egger’s regression of acceptability: in aspect of drop-out rate, Table S1: PRISMA checklist of current meta-analysis, Table S2: Keyword applied in each database and search result, Table S3: Excluded studies and reason, Table S4: characteristics of the included studies, Table S5: (A) SUCRA of the blood transfusion rate; (B) SUCRA of the blood transfusion rate: subgroup of patients with baseline anemia; (C) SUCRA of the improvement in hemoglobin; (D) SUCRA of the improvement in ferritin; (E) SUCRA of the unit of blood transfused; (F) SUCRA of the drop out rate, Table S6: (A) League table of the blood transfusion rate: subgroup of patients with baseline anemia; (B) League table of the improvement in hemoglobin; (C) League table of the improvement in ferritin; (D) League table of the improvement in unit of blood transfused; (E) League table of the drop out rate, Table S7: Inconsistency of different intervention, Table S8: Estimated between-studies standard deviation of different outcome, Table S9: Quality of evidence for primary outcome: rate of blood transfusion.
