# Supplementary material for: Efficacy of Different Interventions to Reduce Pre- or Perioperative Blood Transfusion Rate in Patients with Colorectal Cancer: A Network Meta-Analysis of Randomized Controlled Trials
Source: Curr Oncol. 2021 Aug 22;28(4):3214–26. doi: 10.3390/curroncol28040279 (PMC8395415; doi:10.3390/curroncol28040279)
Supplement: Supplementary file 1 [file curroncol-28-00279-s001.zip › curroncol-1231226-supplementary-figures.pdf]

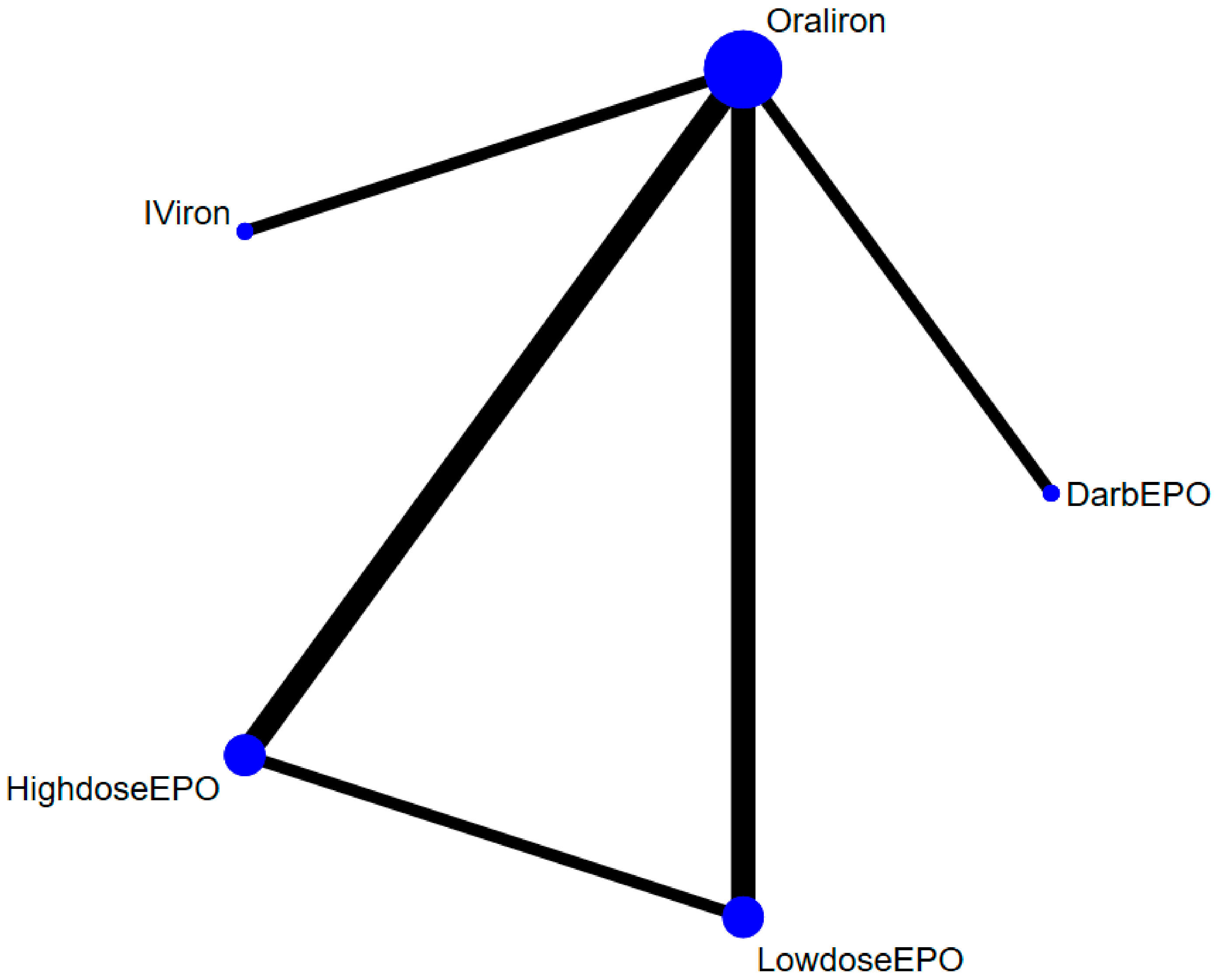

Figure S1A network structure of NMA of rate of blood transfusion: subgroup of baseline anemia

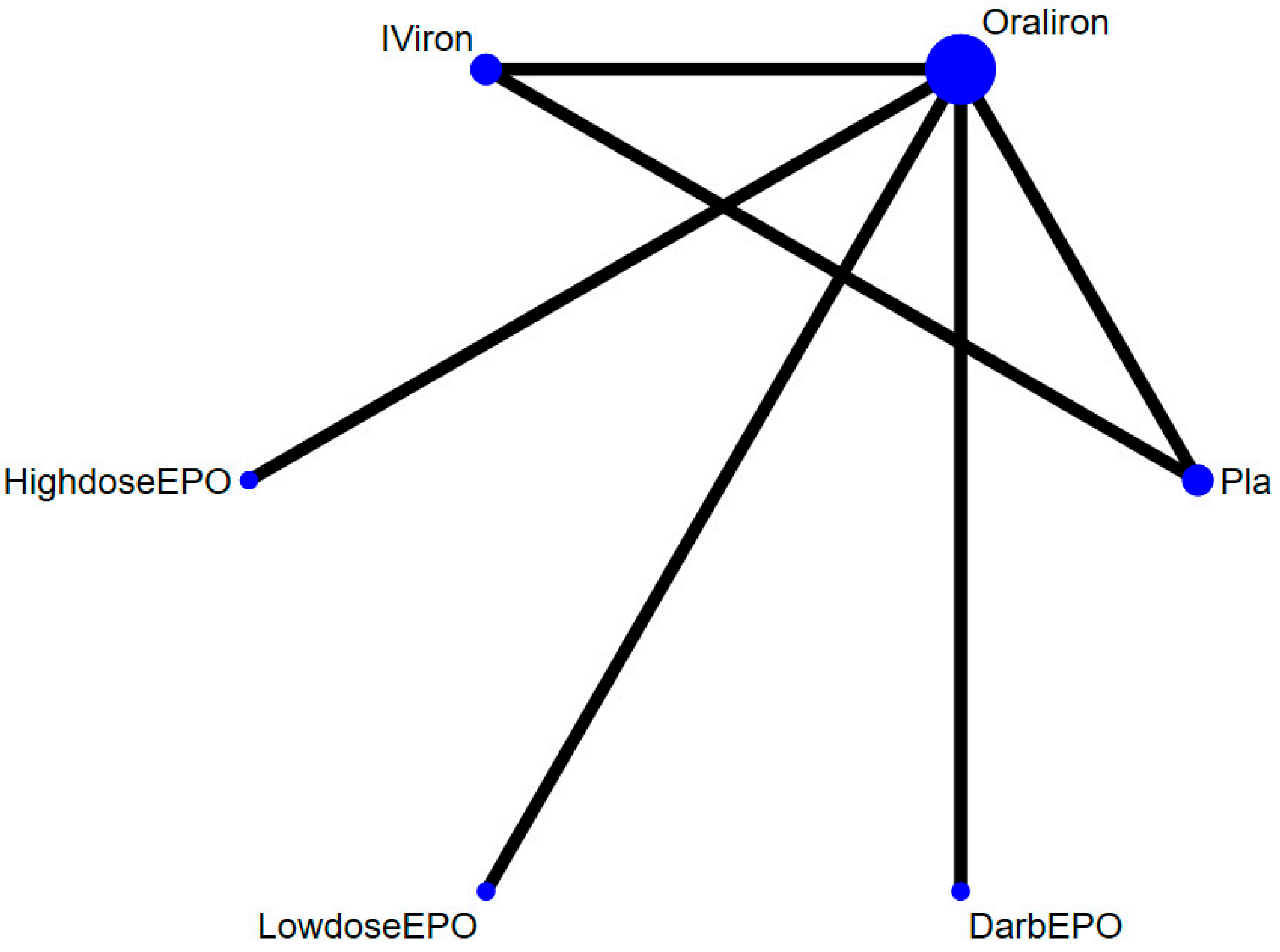

Figure S1B network structure of NMA of changes of hemoglobin

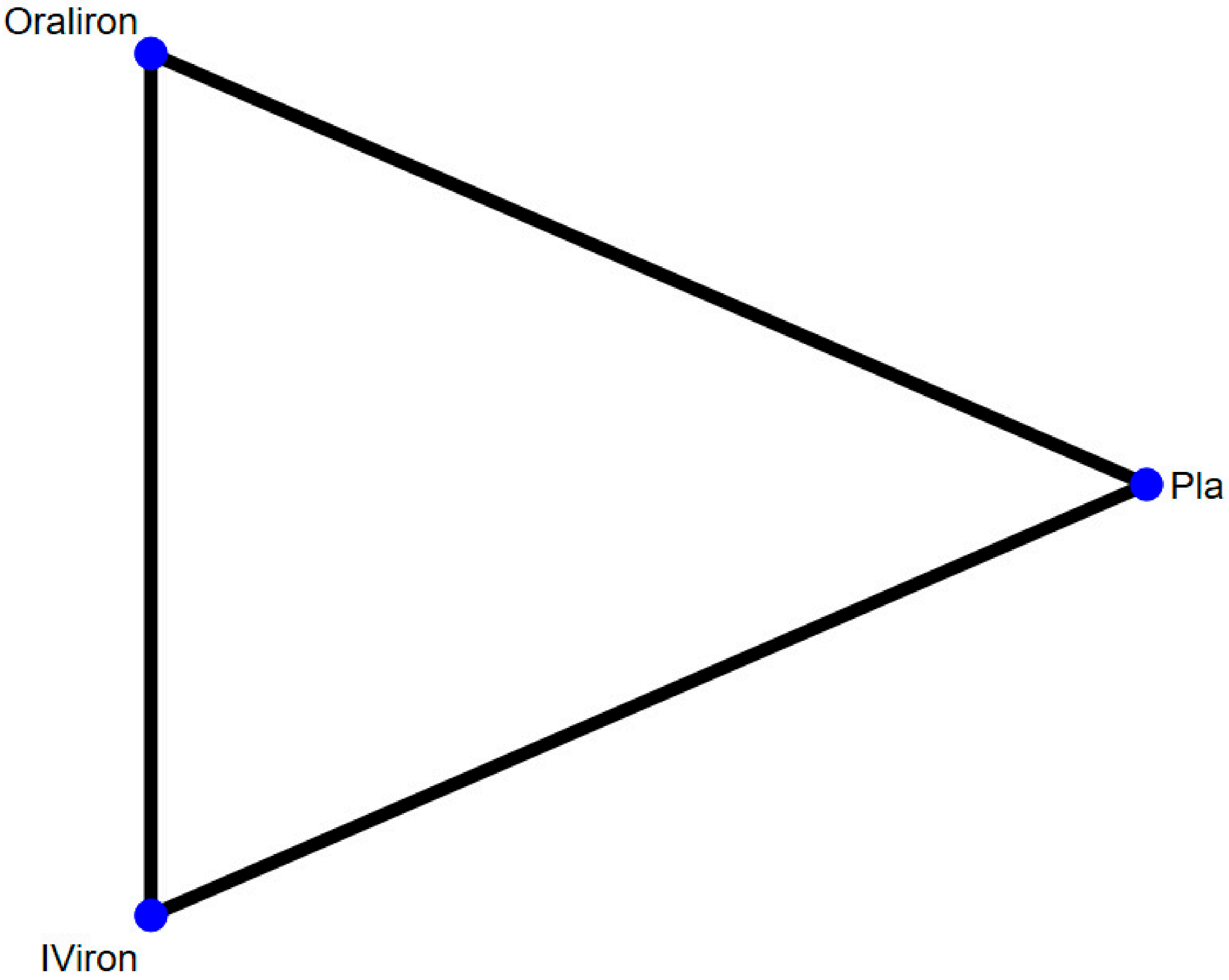

Figure S1C network structure of NMA of changes of ferritin

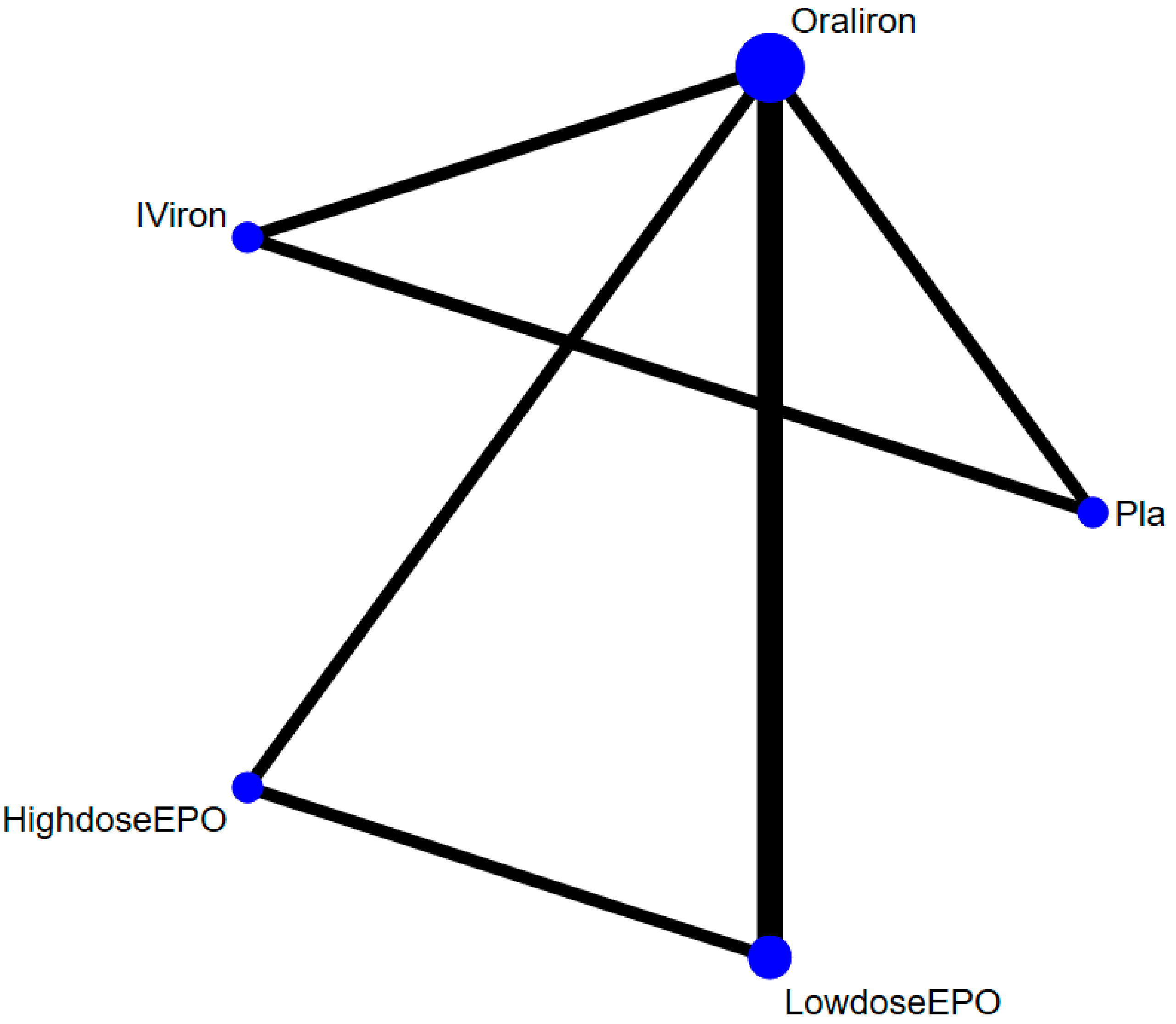

Figure S1D network structure of NMA of amounts of blood transfused

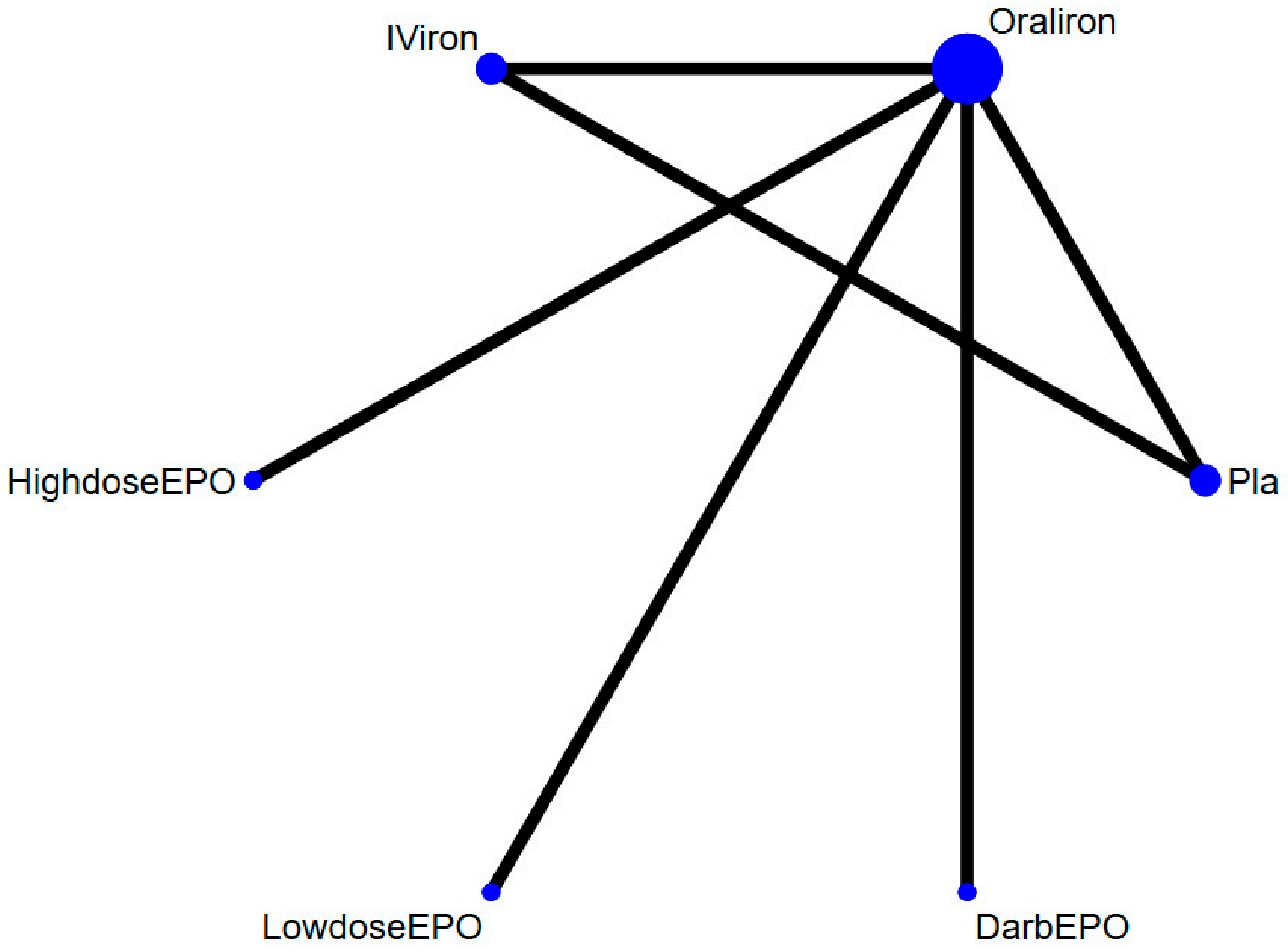

Figure S1E network structure of NMA of acceptability: in aspect of drop-out rate

Figure legend of Figure S1A-S1E

The lines between nodes represent direct comparisons in various trials, and the size of each circle is proportional to the size of the population involved in each specific treatment. The thickness of the lines is proportional to the number of trials connected to the network.

Abbreviation: AMSTAR: assessing the methodological quality of systematic review; CI: confidence interval; DarbEPO: darbepoetin alfa; GRADE: Grading of Recommendations Assessment, Development and Evaluation; HighdoseEPO: epoetin alfa 300 IU/kg; IViron: intravenous iron supplement; LowdoseEPO: epoetin alfa 150 IU/kg; MD: mean difference; NMA: network meta-analysis; OR: odds ratio; Oraliron: oral iron supplement; Pla: placebo/Control; PRISMA: preferred reporting items for systematic reviews and meta-analysis; RCT: randomized controlled trial; SUCRA: surface under the cumulative ranking curve

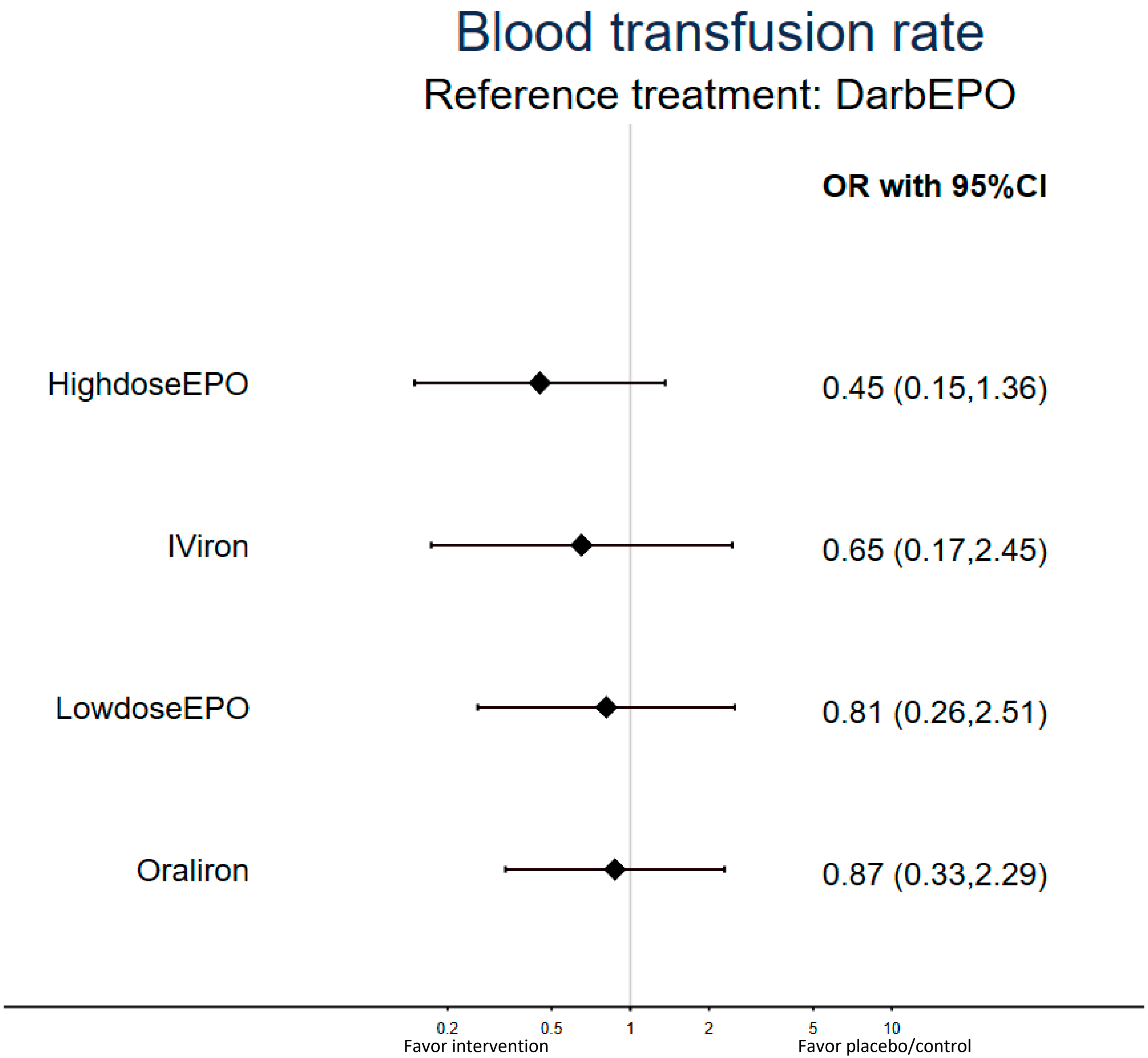

Figure S2A forest plot of NMA of rate of blood transfusion: subgroup of baseline anemia

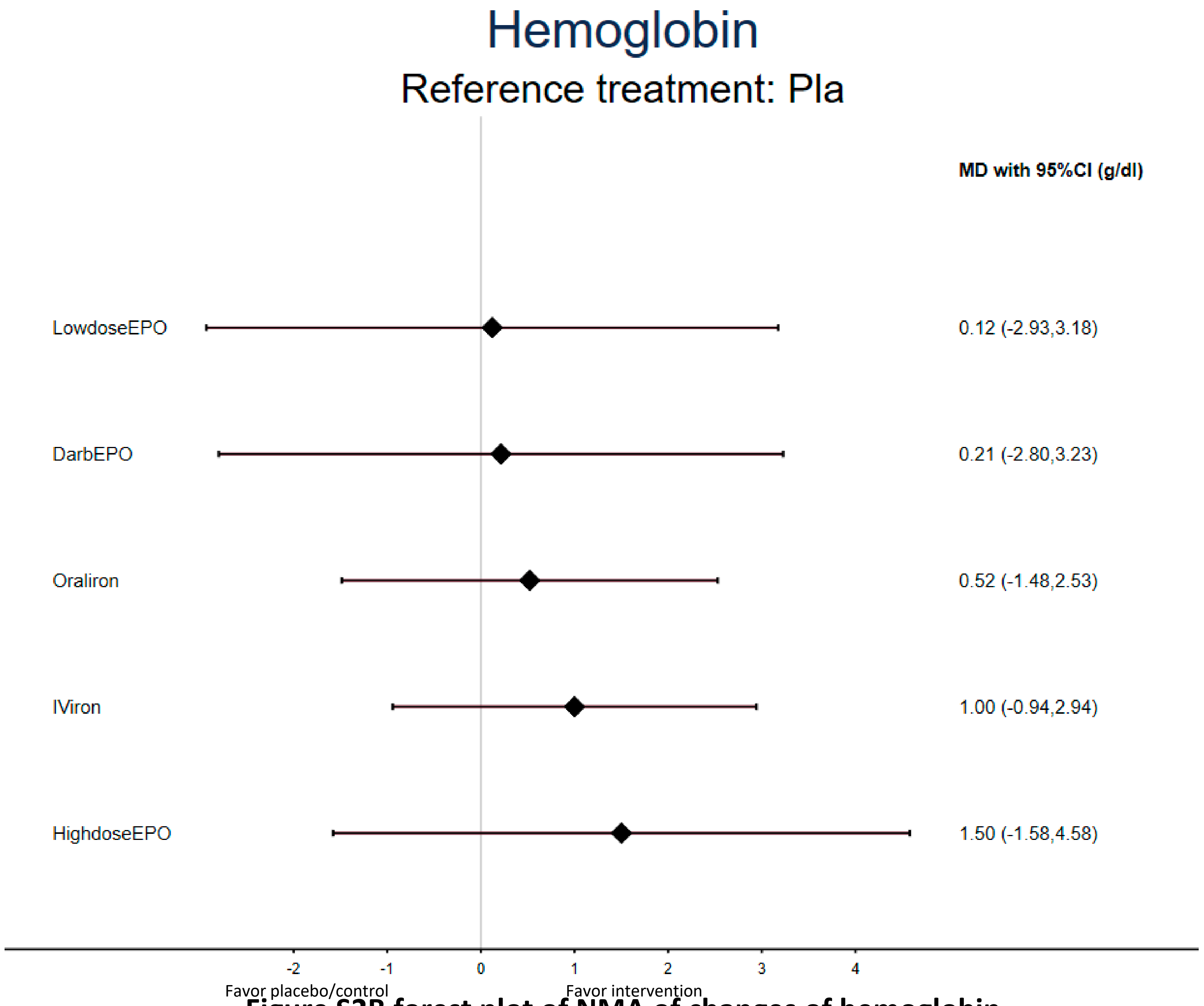

Figure S2B forest plot of NMA of changes of hemoglobin

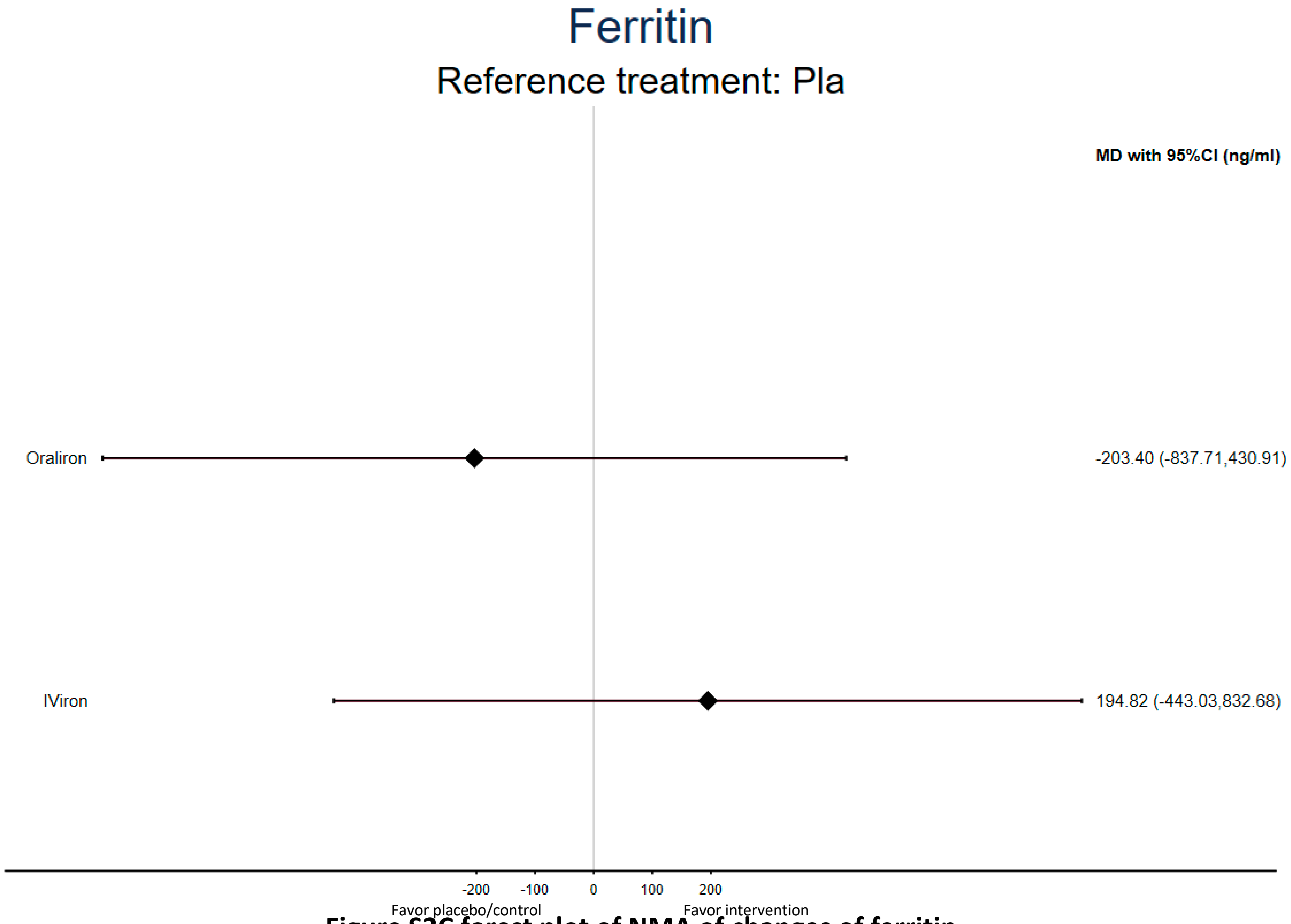

Figure S2C forest plot of NMA of changes of ferritin

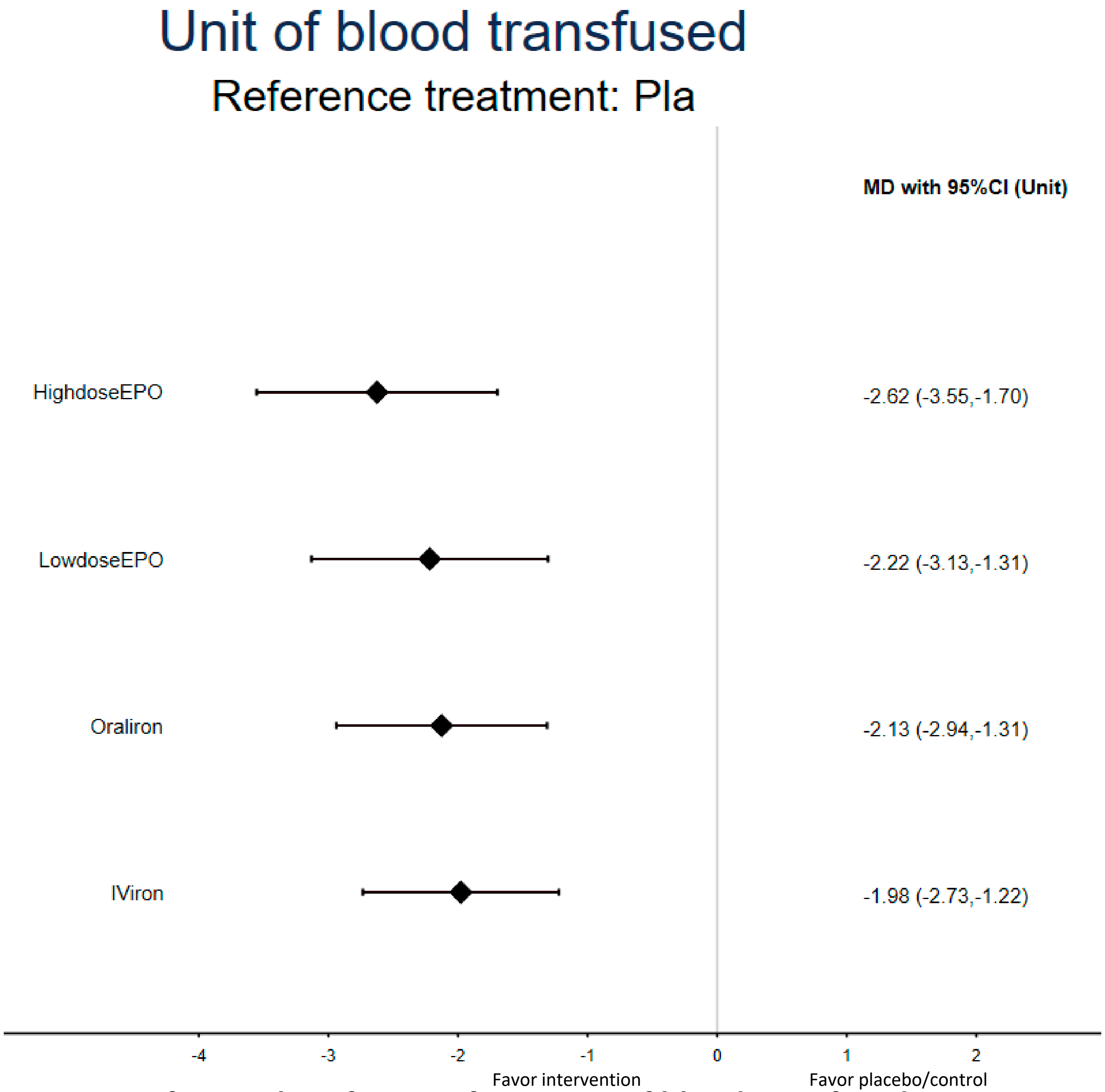

Figure S2D forest plot of NMA of amounts of blood transfused

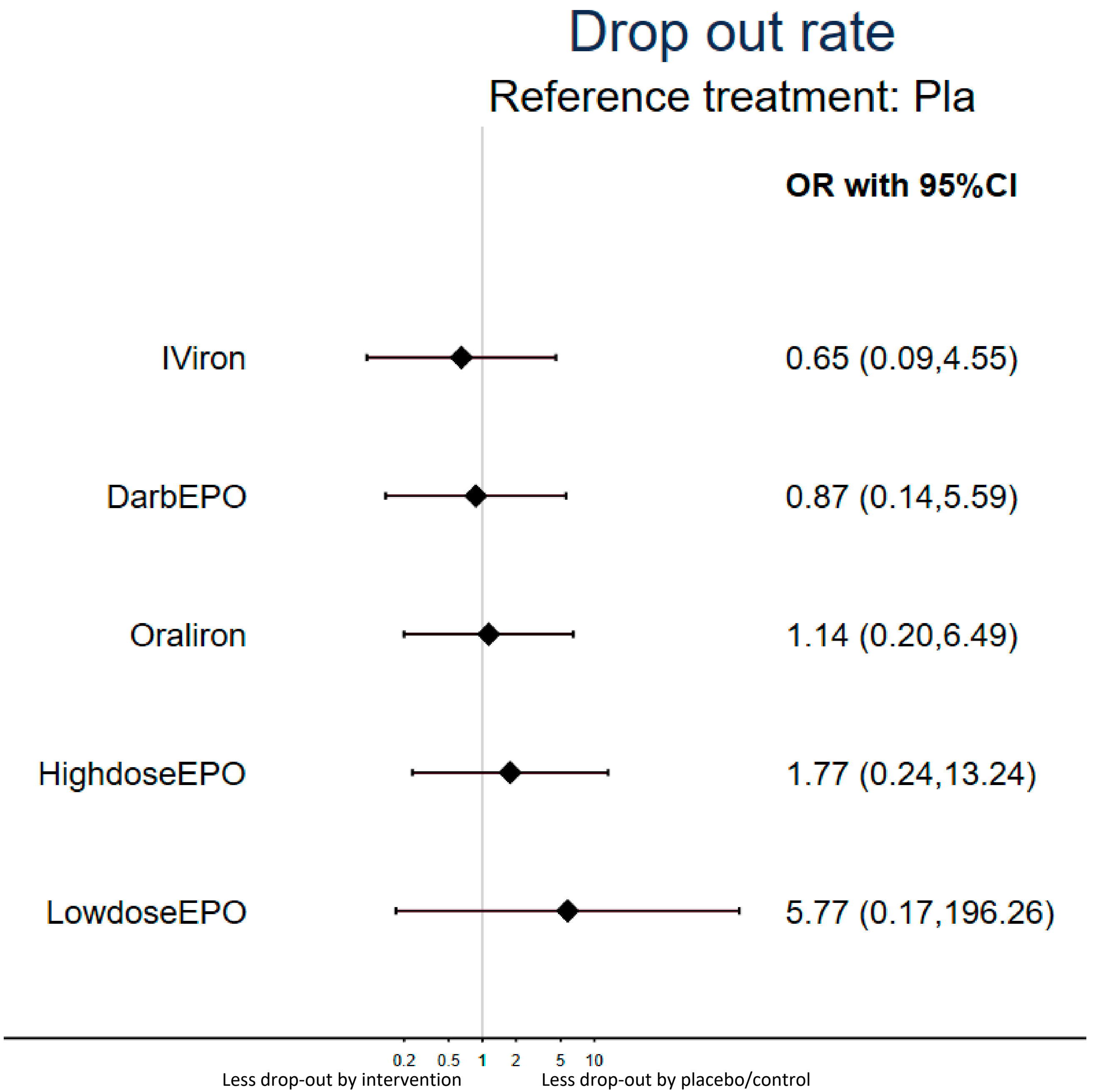

Figure S2E forest plot of NMA of acceptability: in aspect of drop-out rate

Figure legend of Figure S2A-S2E

Abbreviation: AMSTAR: assessing the methodological quality of systematic review; CI: confidence interval; DarbEPO: darbepoetin alfa; GRADE: Grading of Recommendations Assessment, Development and Evaluation; HighdoseEPO: epoetin alfa 300 IU/kg; IViron: intravenous iron supplement; LowdoseEPO: epoetin alfa 150 IU/kg; MD: mean difference; NMA: network meta-analysis; OR: odds ratio; Oraliron: oral iron supplement; Pla: placebo/Control; PRISMA: preferred reporting items for systematic reviews and meta-analysis; RCT: randomized controlled trial; SUCRA: surface under the cumulative ranking curve

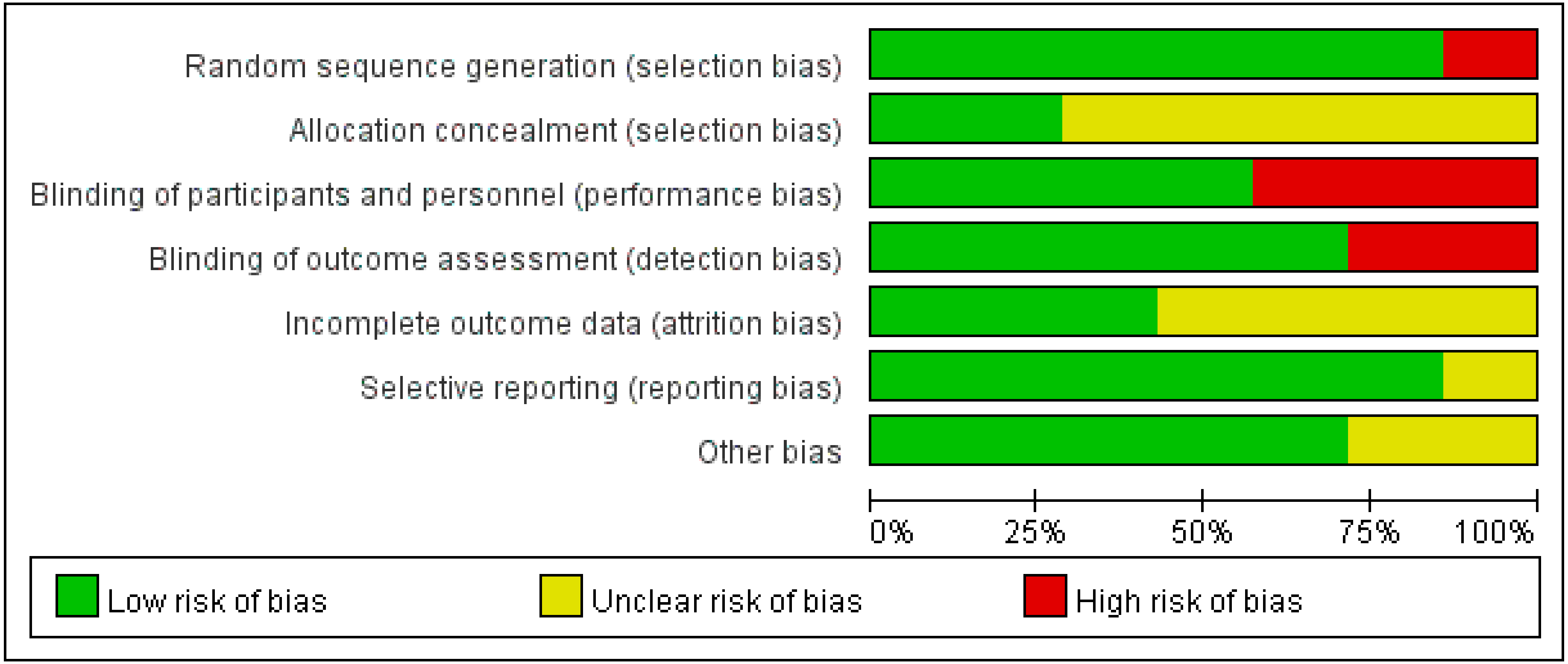

Figure S3A overview of risk of bias

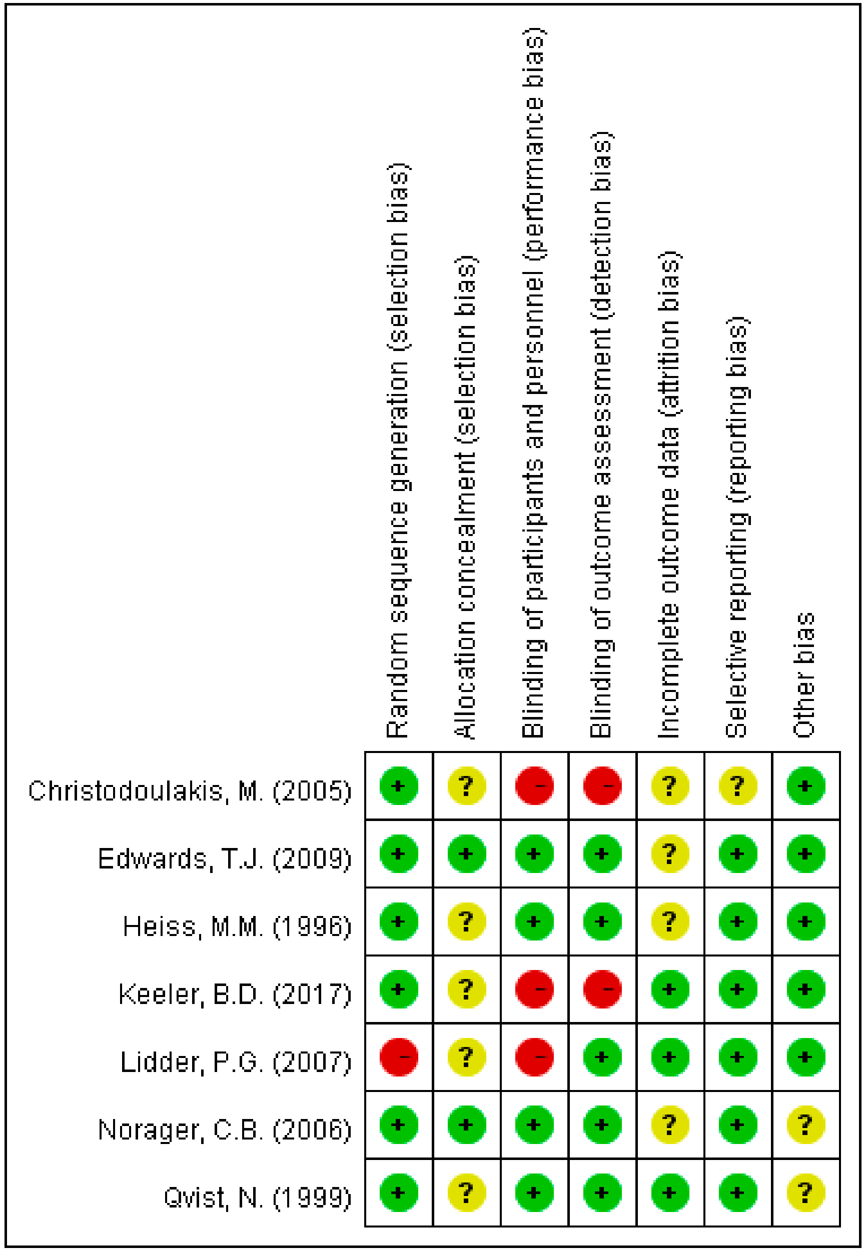

Figure S3B detailed risk of bias in each study

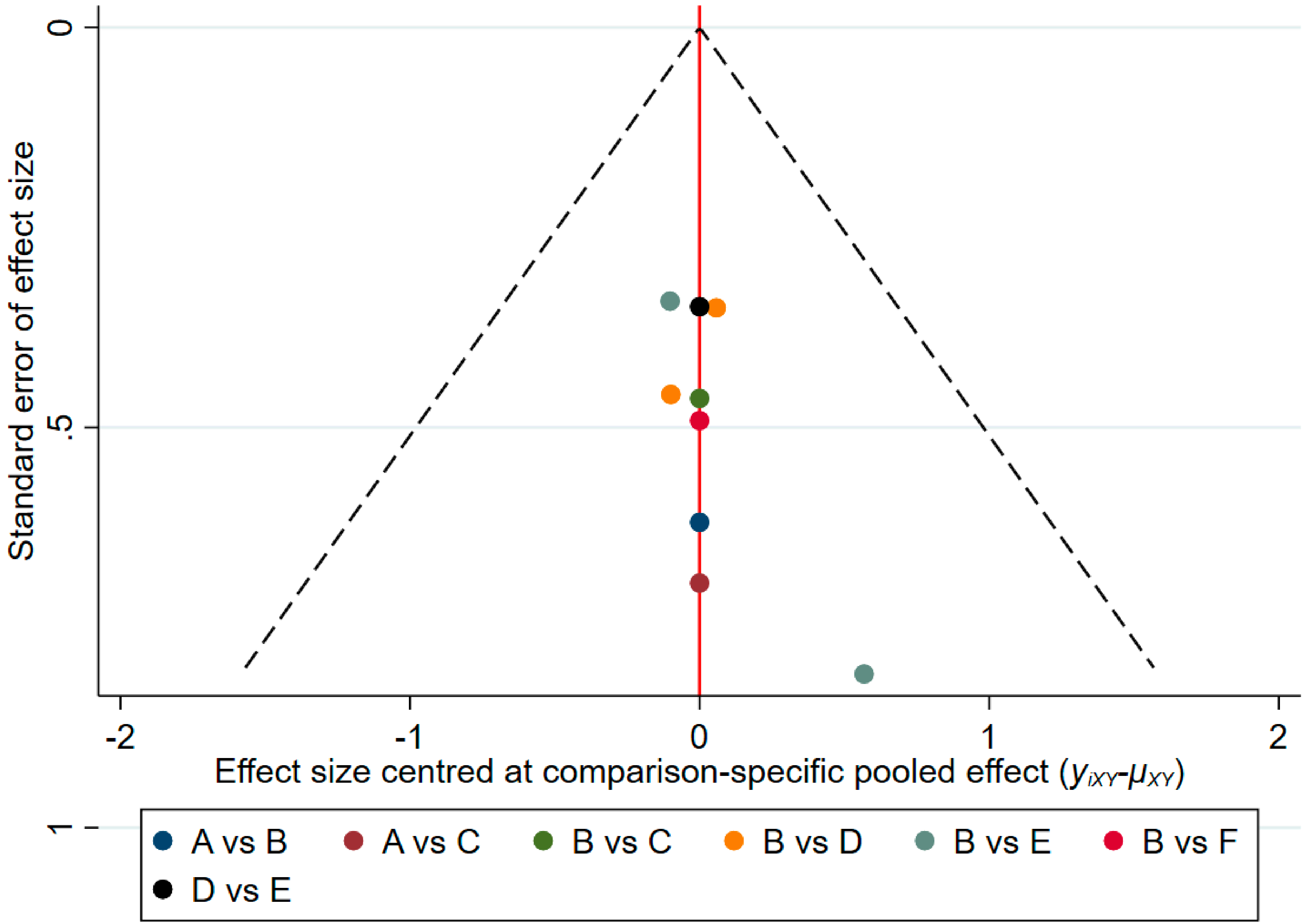

Figure S4A Funnel plot of primary outcome: rate of blood transfusion

Treatments used in Figure S4A

A: Pla

B: Oraliron

C: IViron

D: HighdoseEPO

E: LowdoseEPO

F: DarbEPO

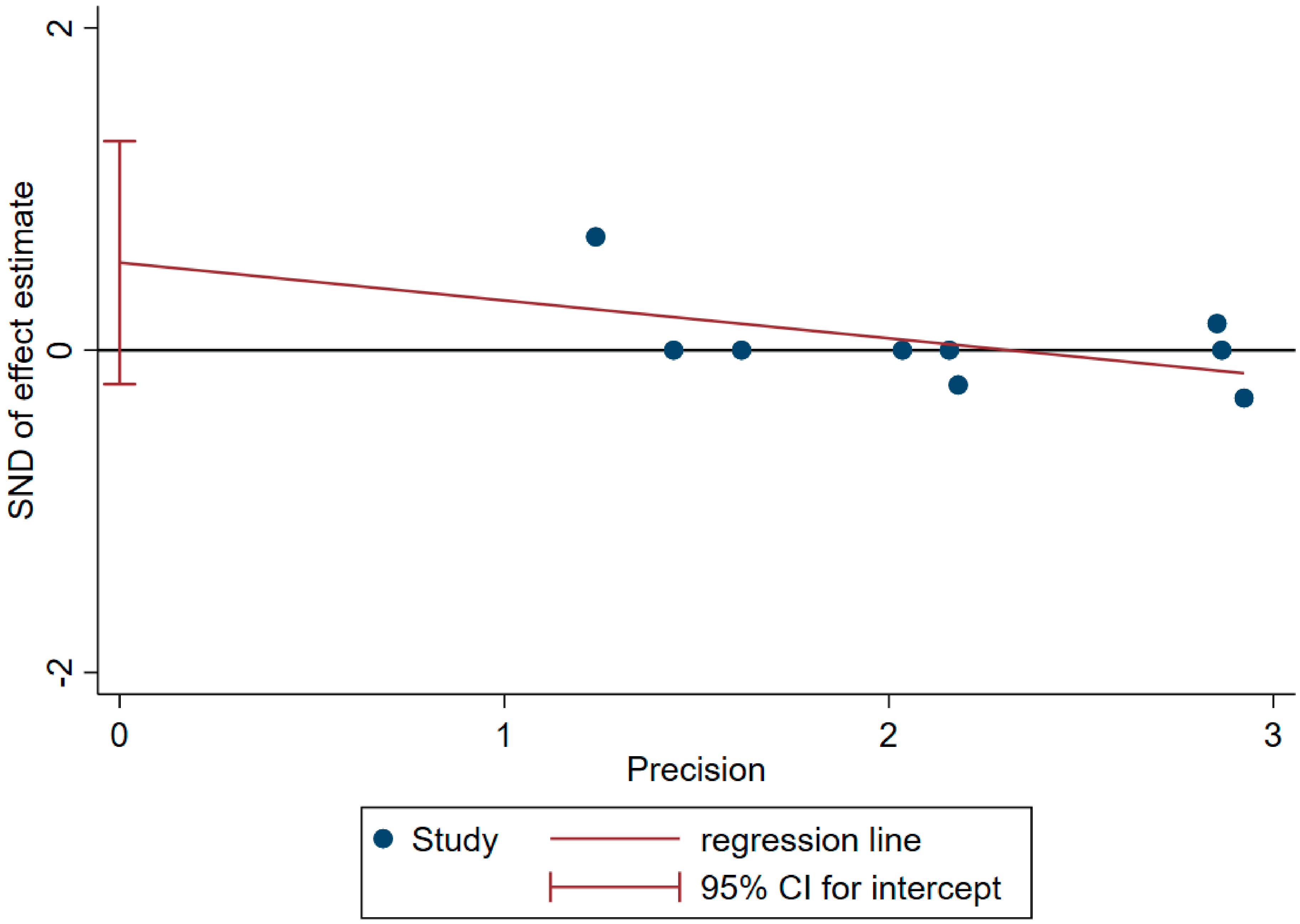

Figure S4B Egger's regression of primary outcome: rate of blood transfusion

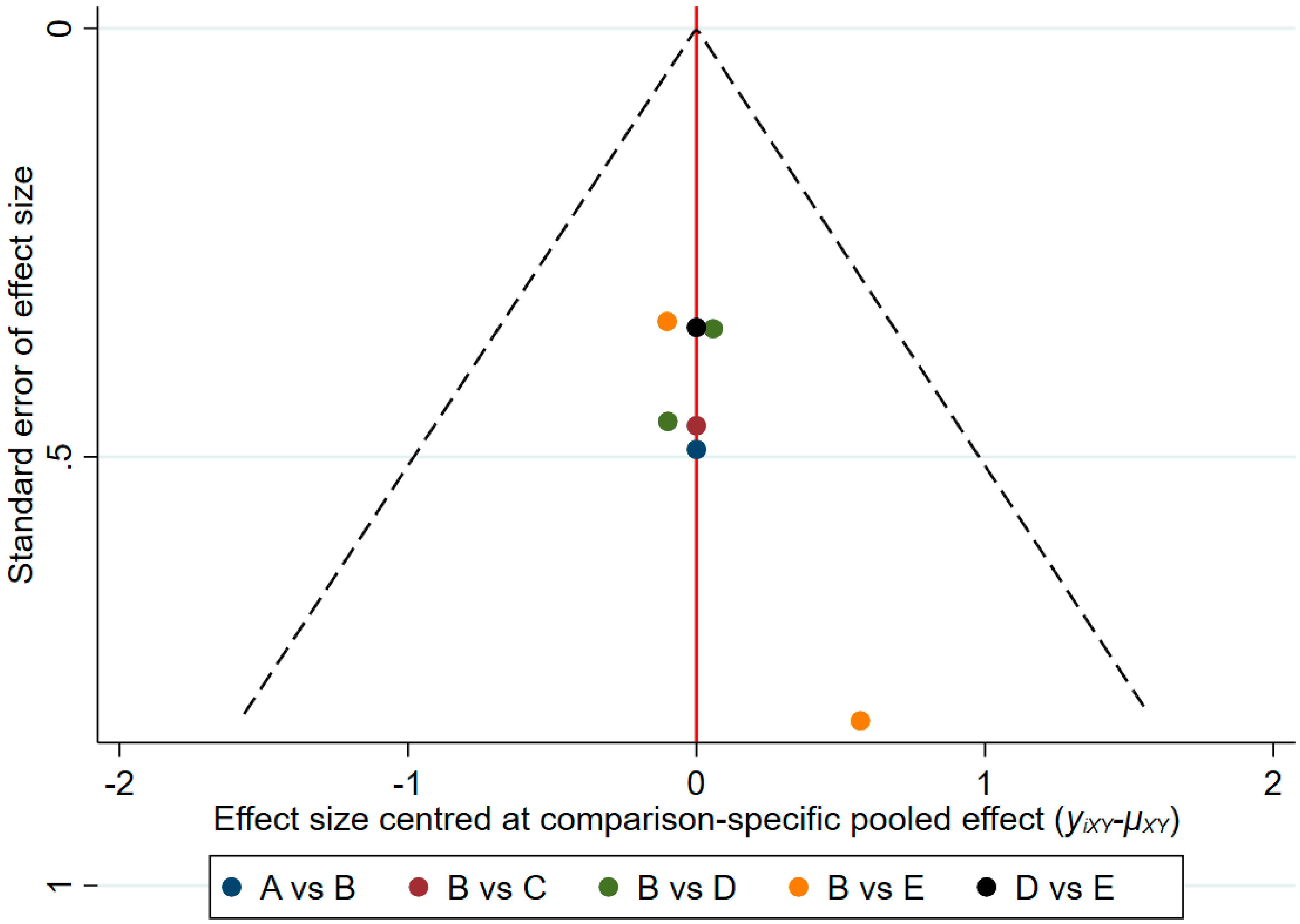

Figure S4C Funnel plot of rate of blood transfusion: subgroup of baseline anemia

Treatments used in Figure S4C

A: DarbEPO

B: Oraliron

C: IViron

D: HighdoseEPO

E: LowdoseEPO

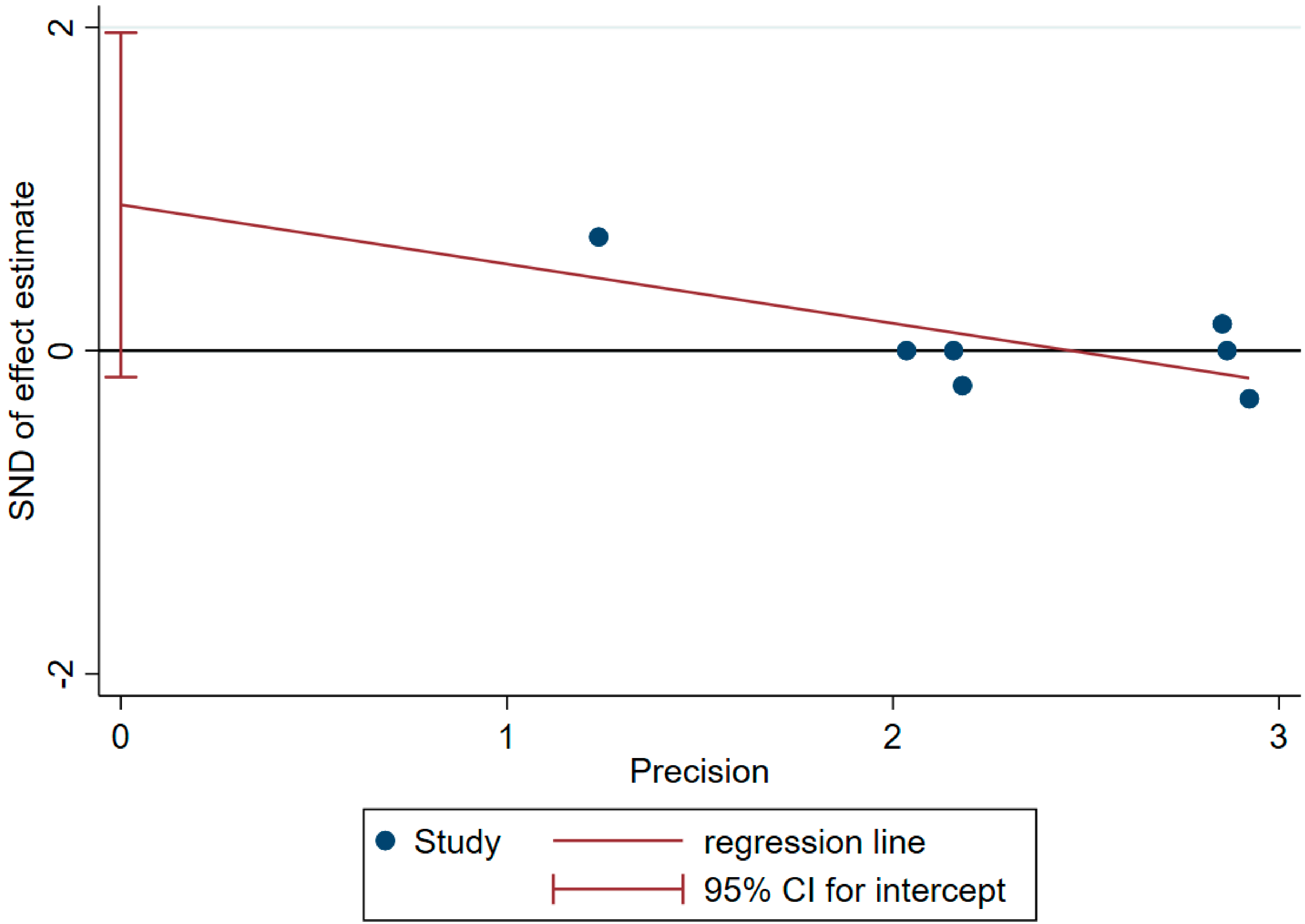

Figure S4D Egger's regression of rate of blood transfusion: subgroup of baseline anemia

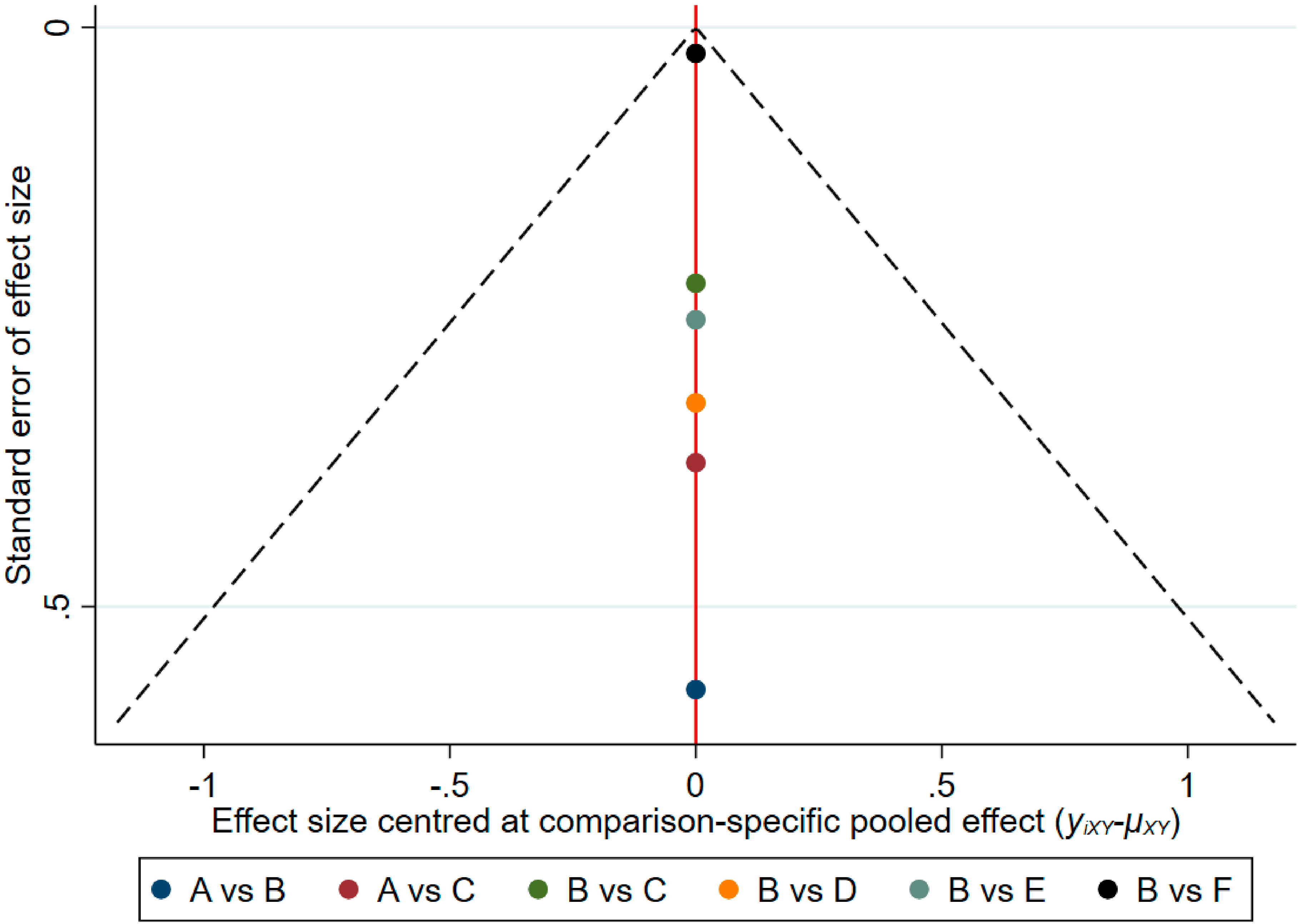

Figure S4E Funnel plot of changes of hemoglobin

Treatments used in Figure S4E

A: Pla

B: Oraliron

C: IViron

D: HighdoseEPO

E: LowdoseEPO

F: DarbEPO

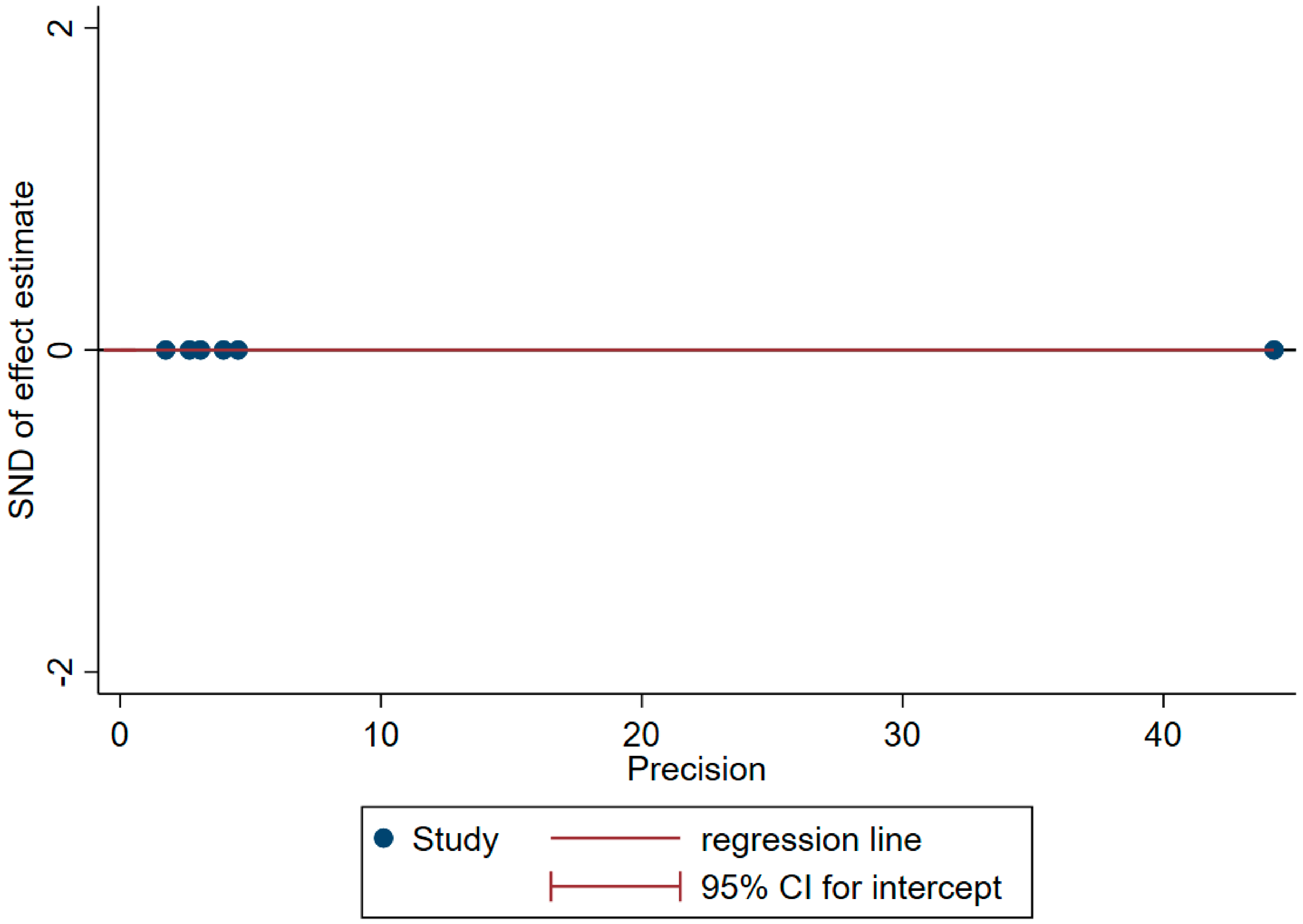

Figure S4F Egger's regression of changes of hemoglobin

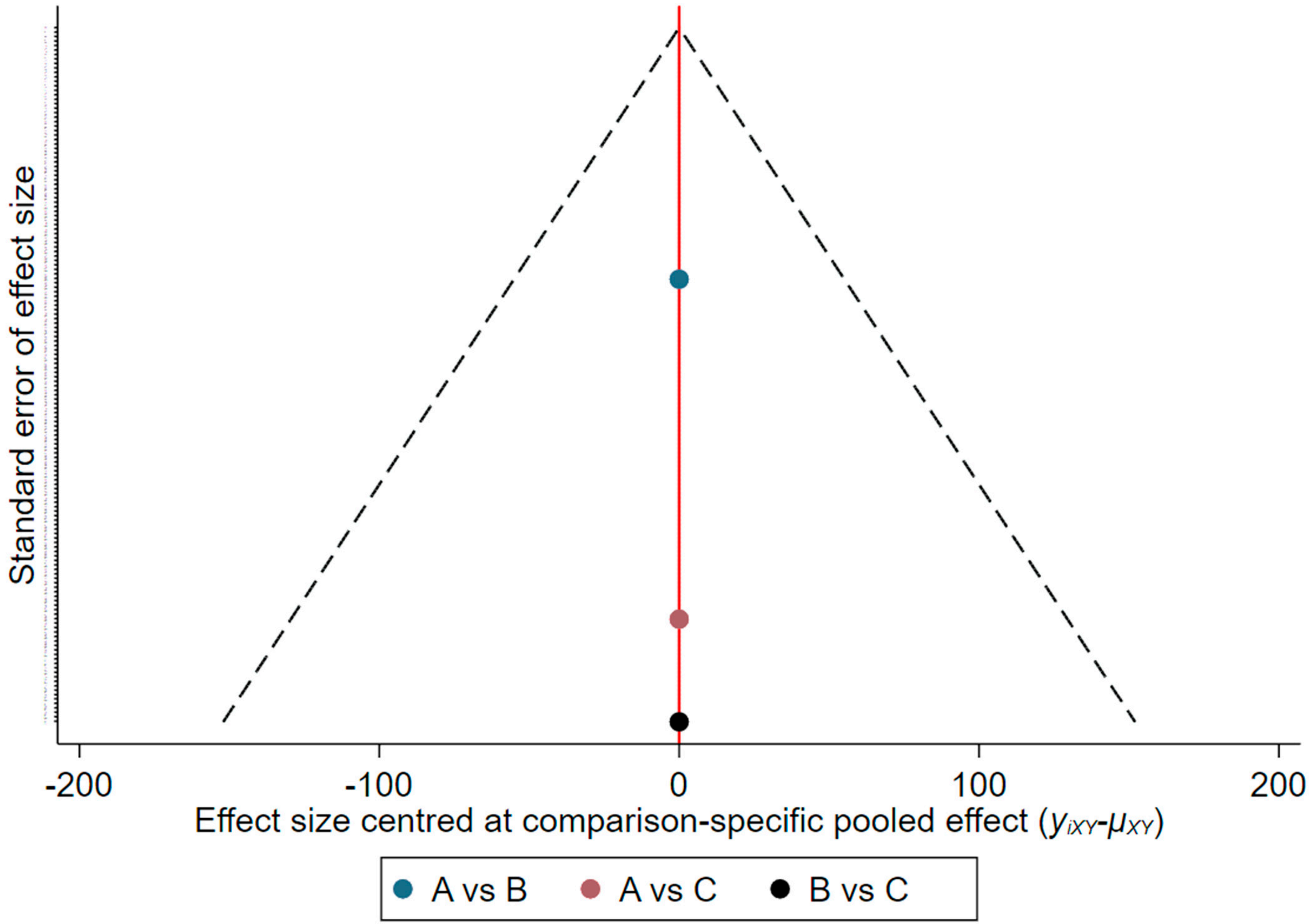

Figure S4G Funnel plot of changes of ferritin

Treatments used in Figure S4G

A: Pla

B: Oraliron

C: IViron

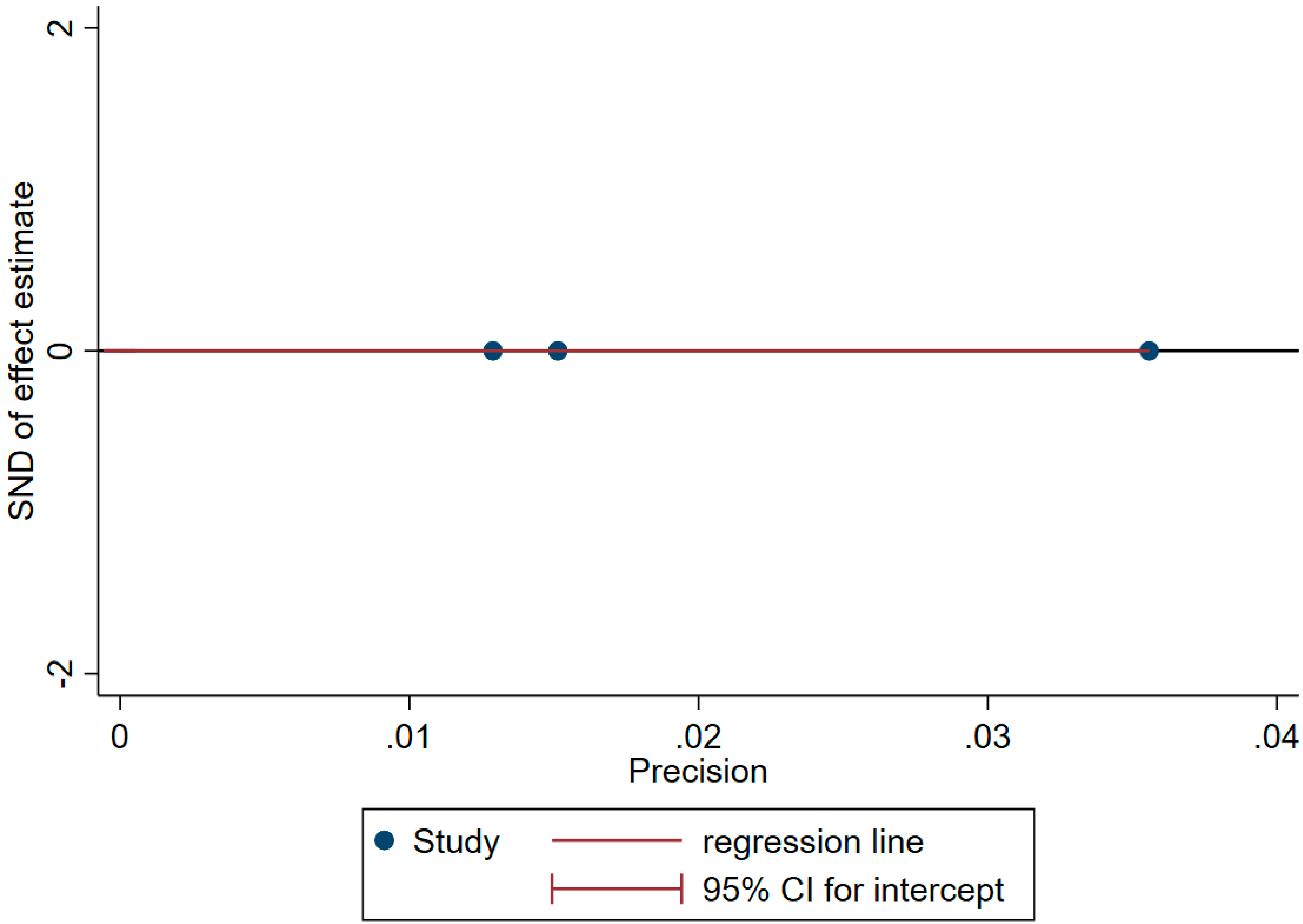

Figure S4H Egger's regression of changes of ferritin

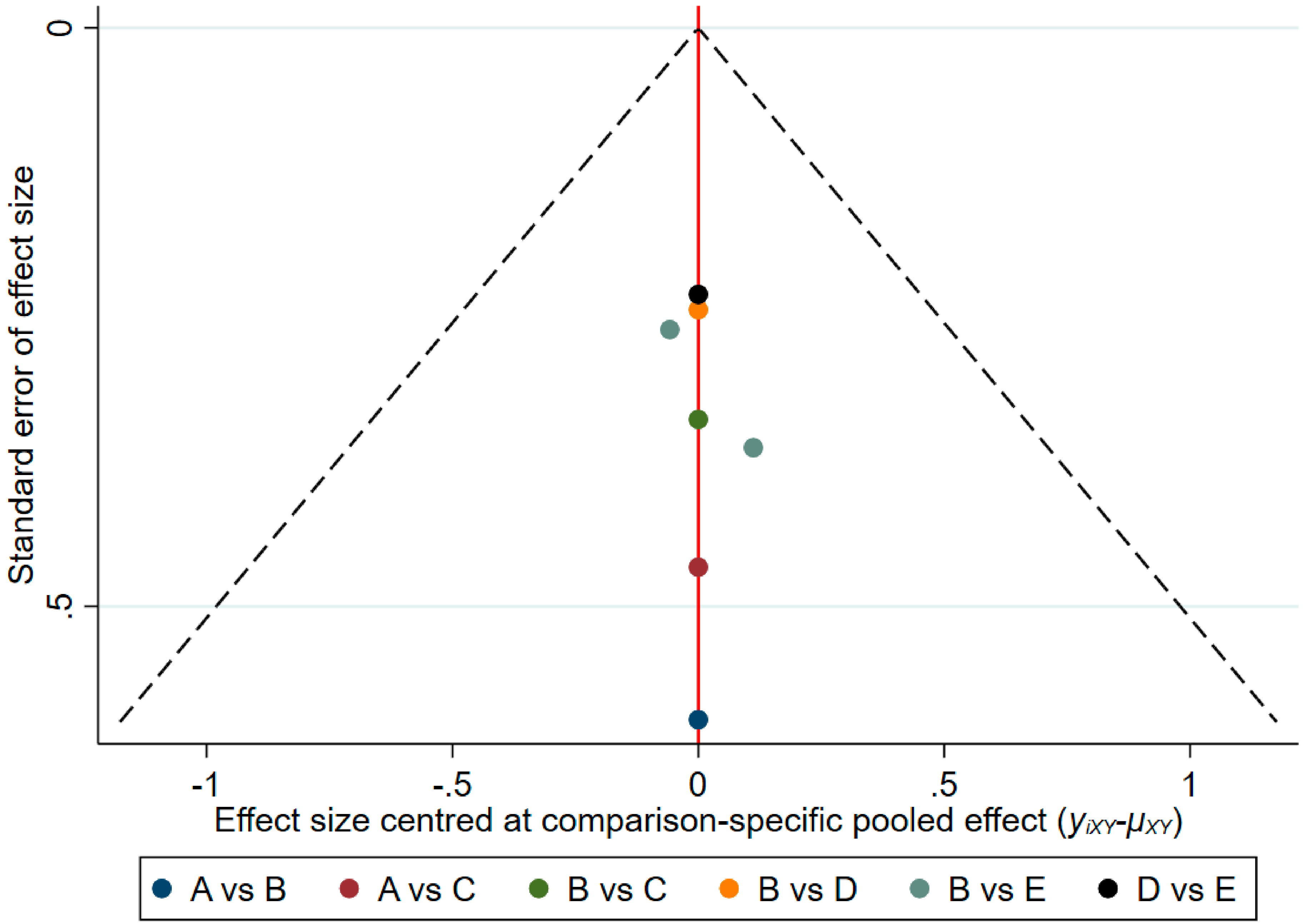

Figure S4I Funnel plot of amounts of blood transfused

Treatments used in Figure S4I

A: Pla
B: Oraliron
C: IViron
D: HighdoseEPO
E: LowdoseEPO

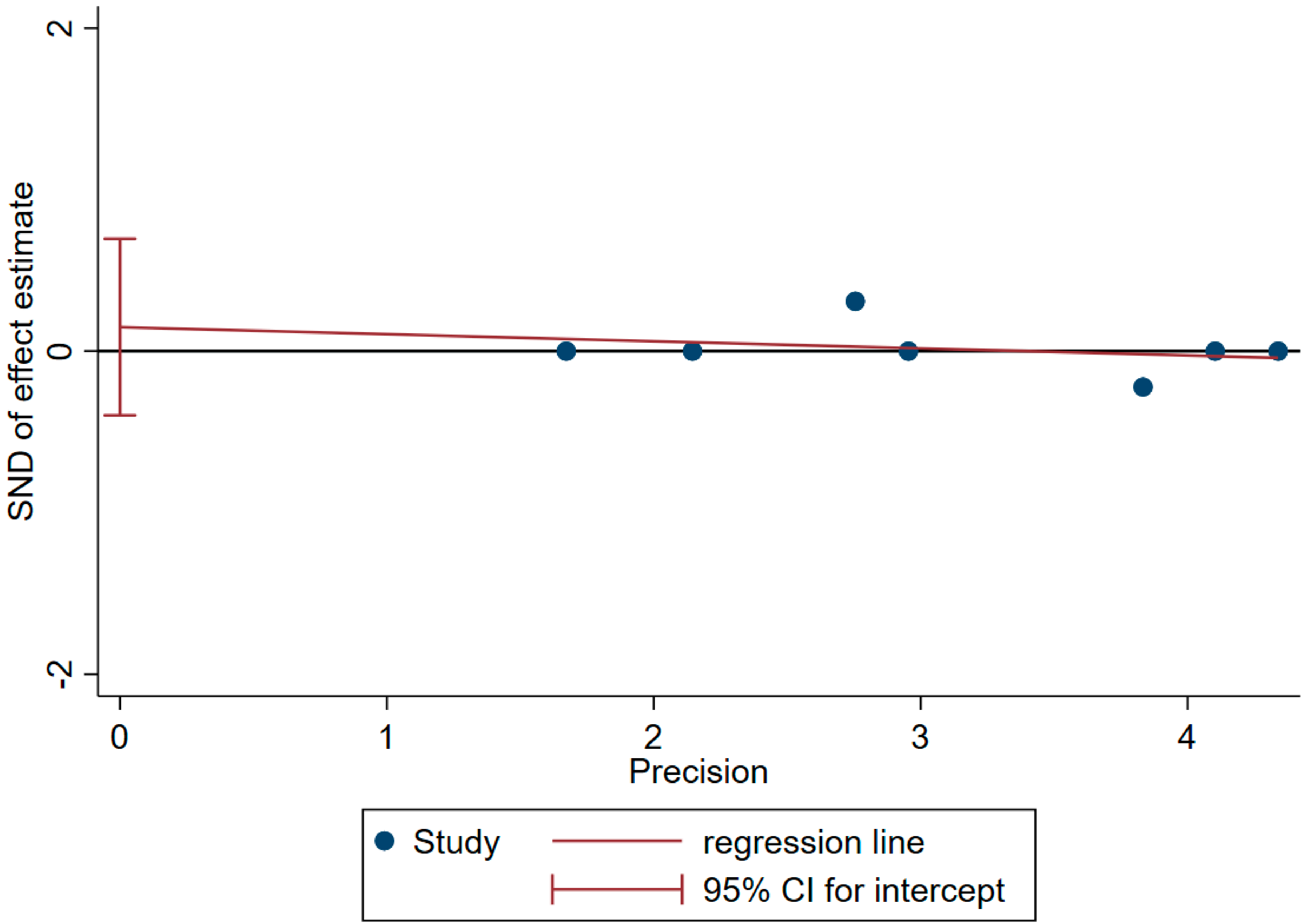

Figure S4J Egger's regression of amounts of blood transfused

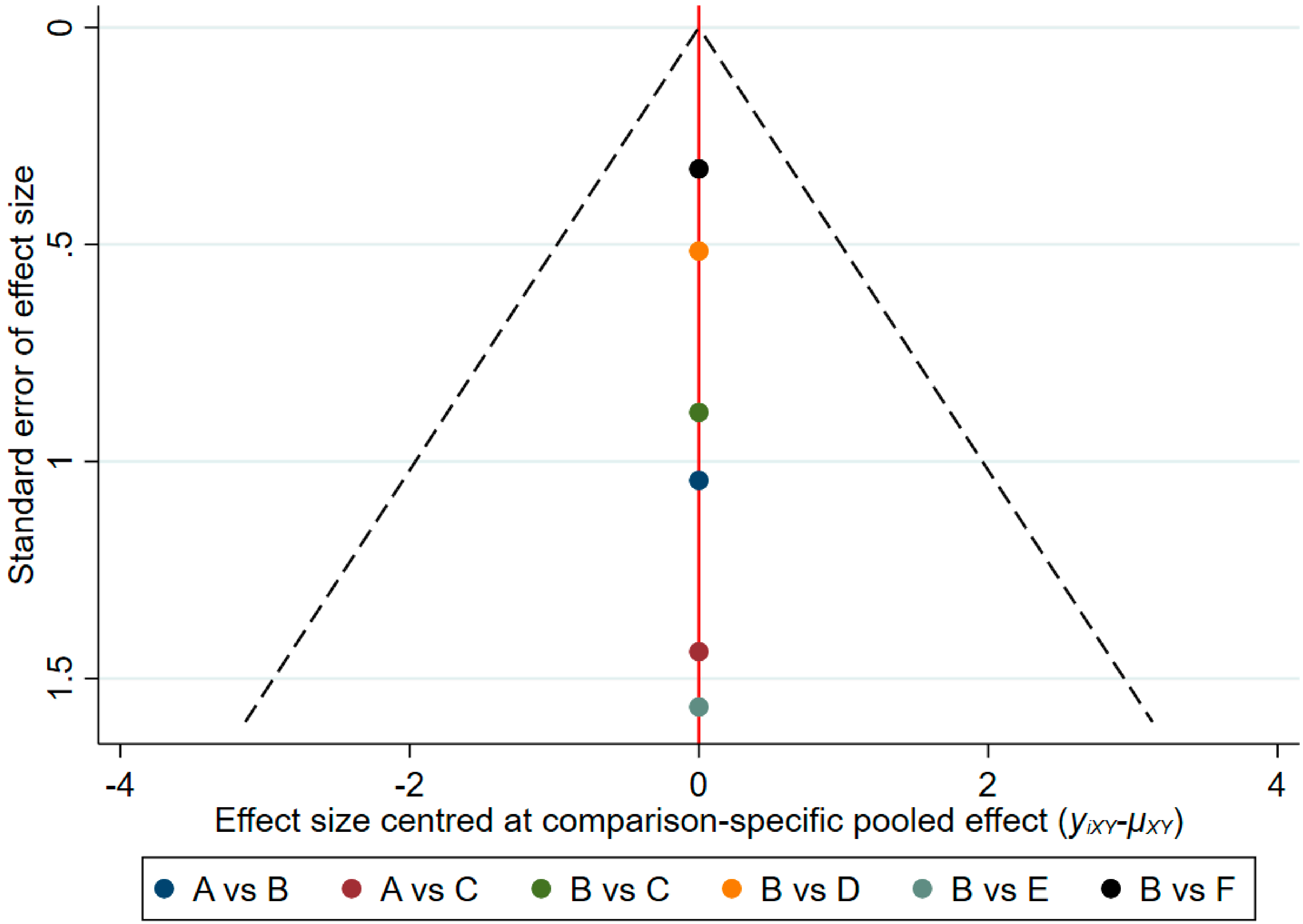

Figure S4K Funnel plot of acceptability: in aspect of drop-out rate

Treatments used in Figure S4K

A: Pla

B: Oraliron

C: IViron

D: HighdoseEPO

E: LowdoseEPO

F: DarbEPO

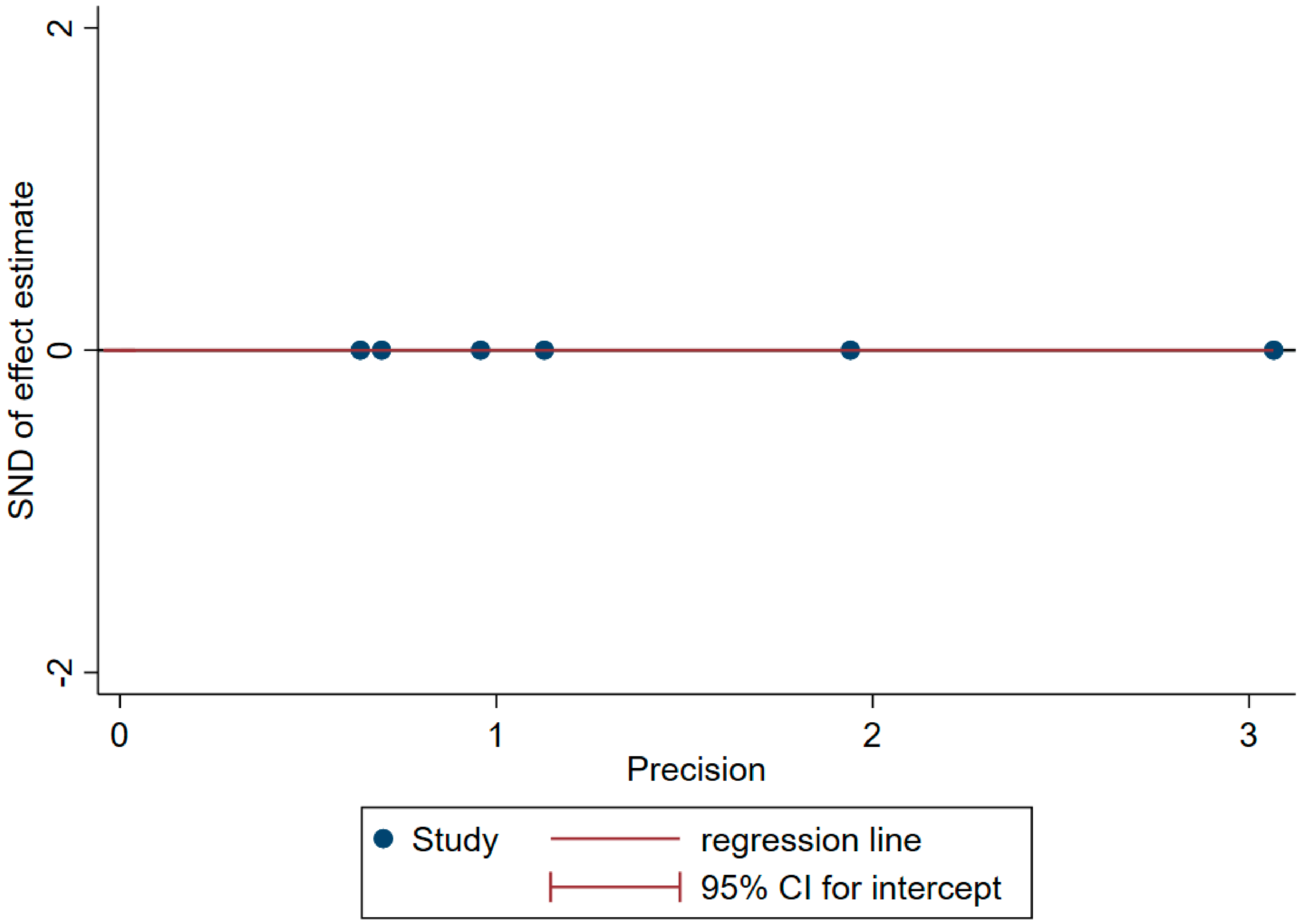

Figure S4L Egger’s regression of acceptability: in aspect of drop-out rate

Figure legend of Figure S4A-S4L

Abbreviation: AMSTAR: assessing the methodological quality of systematic review; CI: confidence interval; DarbEPO: darbepoetin alfa; GRADE: Grading of Recommendations Assessment, Development and Evaluation; HighdoseEPO: epoetin alfa 300 IU/kg; IViron: intravenous iron supplement; LowdoseEPO: epoetin alfa 150 IU/kg; MD: mean difference; NMA: network meta-analysis; OR: odds ratio; Oraliron: oral iron supplement; Pla: placebo/Control; PRISMA: preferred reporting items for systematic reviews and meta-analysis; RCT: randomized controlled trial; SUCRA: surface under the cumulative ranking curve
