# Supplementary material for: Efficacy of Different Interventions to Reduce Pre- or Perioperative Blood Transfusion Rate in Patients with Colorectal Cancer: A Network Meta-Analysis of Randomized Controlled Trials
Source: Curr Oncol. 2021 Aug 22;28(4):3214–26. doi: 10.3390/curroncol28040279 (PMC8395415; doi:10.3390/curroncol28040279)
Supplement: Supplementary file 1 [file curroncol-28-00279-s001.zip › curroncol-1231226-supplementary-tables.pdf]

Table S1: PRISMA checklist of current meta-analysis

Section and Topic	Item #	Checklist item	Page where item is reported
**TITLE**			
Title	1	Identify the report as a systematic review.	1
**ABSTRACT**			
Abstract	2	See the PRISMA 2020 for Abstracts checklist.	4
**INTRODUCTION**			
Rationale	3	Describe the rationale for the review in the context of existing knowledge.	5-6
Objectives	4	Provide an explicit statement of the objective(s) or question(s) the review addresses.	5-6
**METHODS**			
Eligibility criteria	5	Specify the inclusion and exclusion criteria for the review and how studies were grouped for the syntheses.	7-8
Information sources	6	Specify all databases, registers, websites, organisations, reference lists and other sources searched or consulted to identify studies. Specify the date when each source was last searched or consulted.	7-8
Search strategy	7	Present the full search strategies for all databases, registers and websites, including any filters and limits used.	7-8
Selection process	8	Specify the methods used to decide whether a study met the inclusion criteria of the review, including how many reviewers screened each record and each report retrieved, whether they worked independently, and if applicable, details of automation tools used in the process.	7-8
Data collection process	9	Specify the methods used to collect data from reports, including how many reviewers collected data from each report, whether they worked independently, any processes for obtaining or confirming data from study investigators, and if applicable, details of automation tools used in the process.	7-8
Data items	10a	List and define all outcomes for which data were sought. Specify whether all results that were compatible with each outcome domain in each study were sought (e.g. for all measures, time points, analyses), and if not, the methods used to decide which results to collect.	7-8
	10b	List and define all other variables for which data were sought (e.g. participant and intervention characteristics, funding sources). Describe any assumptions made about any missing or unclear information.	7-8
Study risk of bias assessment	11	Specify the methods used to assess risk of bias in the included studies, including details of the tool(s) used, how many reviewers assessed each study and whether they worked independently, and if applicable, details of automation tools used in the process.	8-9
Effect measures	12	Specify for each outcome the effect measure(s) (e.g. risk ratio, mean difference) used in the synthesis or presentation of results.	8-9
Synthesis methods	13a	Describe the processes used to decide which studies were eligible for each synthesis (e.g. tabulating the study intervention characteristics and comparing against the planned groups for each synthesis (item #5)).	8-9
	13b	Describe any methods required to prepare the data for presentation or synthesis, such as handling of missing summary statistics, or data conversions.	8-9
	13c	Describe any methods used to tabulate or visually display results of individual studies and syntheses.	8-9
	13d	Describe any methods used to synthesize results and provide a rationale for the choice(s). If meta-analysis was performed, describe the model(s), method(s) to identify the presence and extent of statistical heterogeneity, and software package(s) used.	8-9
	13e	Describe any methods used to explore possible causes of heterogeneity among study results (e.g. subgroup analysis, meta-regression).	9-10
	13f	Describe any sensitivity analyses conducted to assess robustness of the synthesized results.	9-10
Reporting bias assessment	14	Describe any methods used to assess risk of bias due to missing results in a synthesis (arising from reporting biases).	9-10
Certainty assessment	15	Describe any methods used to assess certainty (or confidence) in the body of evidence for an outcome.	9-10
**RESULTS**			

Section and Topic	Item #	Checklist item	Page where item is reported
Study selection	16a	Describe the results of the search and selection process, from the number of records identified in the search to the number of studies included in the review, ideally using a flow diagram.	11-12, Fig 1, eTab 2
	16b	Cite studies that might appear to meet the inclusion criteria, but which were excluded, and explain why they were excluded.	11-12, eTab 3
Study characteristics	17	Cite each included study and present its characteristics.	11-12, eTab 4
Risk of bias in studies	18	Present assessments of risk of bias for each included study.	11-12, eFig 3
Results of individual studies	19	For all outcomes, present, for each study: (a) summary statistics for each group (where appropriate) and (b) an effect estimate and its precision (e.g. confidence/credible interval), ideally using structured tables or plots.	12-13, eTab 4
Results of syntheses	20a	For each synthesis, briefly summarise the characteristics and risk of bias among contributing studies.	12-13, Fig 2
	20b	Present results of all statistical syntheses conducted. If meta-analysis was done, present for each the summary estimate and its precision (e.g. confidence/credible interval) and measures of statistical heterogeneity. If comparing groups, describe the direction of the effect.	12-13, Fig 3
	20c	Present results of all investigations of possible causes of heterogeneity among study results.	12-13, eTab 7-8
	20d	Present results of all sensitivity analyses conducted to assess the robustness of the synthesized results.	12-15
Reporting biases	21	Present assessments of risk of bias due to missing results (arising from reporting biases) for each synthesis assessed.	12-15, eFig 4
Certainty of evidence	22	Present assessments of certainty (or confidence) in the body of evidence for each outcome assessed.	14-16, eTab 9
**DISCUSSION**			
Discussion	23a	Provide a general interpretation of the results in the context of other evidence.	17-18
	23b	Discuss any limitations of the evidence included in the review.	19
	23c	Discuss any limitations of the review processes used.	19
	23d	Discuss implications of the results for practice, policy, and future research.	20
**OTHER INFORMATION**			
Registration and protocol	24a	Provide registration information for the review, including register name and registration number, or state that the review was not registered.	4
	24b	Indicate where the review protocol can be accessed, or state that a protocol was not prepared.	4
	24c	Describe and explain any amendments to information provided at registration or in the protocol.	4
Support	25	Describe sources of financial or non-financial support for the review, and the role of the funders or sponsors in the review.	21
Competing interests	26	Declare any competing interests of review authors.	21
Availability of data, code and other materials	27	Report which of the following are publicly available and where they can be found: template data collection forms; data extracted from included studies; data used for all analyses; analytic code; any other materials used in the review.	21

The current checklist followed the latest PRISMA 2020 guideline [1].

Reference

1. Page, M.J.; McKenzie, J.E.; Bossuyt, P.M.; Boutron, I.; Hoffmann, T.C.; Mulrow, C.D.; Shamseer, L.; Tetzlaff, J.M.; Akl, E.A.; Brennan, S.E.; et al. The PRISMA 2020 statement: an updated guideline for reporting systematic reviews. *Bmj* **2021**, *372*, n71, doi:10.1136/bmj.n71.

Table S2: Keyword applied in each database and search result

Database	Keyword	Filter	Date	Result
PubMed	(iron OR ferritin OR ferric OR ferrous OR erythropoietin OR epoetin alfa OR EPO OR haemopoietin OR haemopoietic OR epoetin beta OR haematopoietin OR erythrogenin OR erythrogenic OR erythropoetin) AND (colorectal cancer OR colorectal neoplasm OR colorectal carcinoma OR colorectal tumor OR colon cancer OR colon neoplasm OR rectal cancer OR rectum cancer OR rectal neoplasm OR rectum neoplasm OR colon tumor OR rectum tumor OR rectal tumor) AND (random OR randomized OR randomised)	N/A	2021/4/25	100
ClinicalKey	(iron OR erythropoietin) AND (colorectal cancer) AND (random OR randomized OR randomised)	N/A	2021/4/25	7
Cochrane CENTRAL	(iron OR ferritin OR ferric OR ferrous OR erythropoietin OR epoetin alfa OR EPO OR haemopoietin OR haemopoietic OR epoetin beta OR haematopoietin OR erythrogenin OR erythrogenic OR erythropoetin) AND (colorectal cancer OR colorectal neoplasm OR colorectal carcinoma OR colorectal tumor OR colon cancer OR colon neoplasm OR rectal cancer OR rectum cancer OR rectal neoplasm OR rectum neoplasm OR colon tumor OR rectum tumor OR rectal tumor) AND (random OR randomized OR randomised)	N/A	2021/4/25	112
Embase	(iron OR ferritin OR ferric OR ferrous) AND (colorectal cancer OR colorectal neoplasm OR colorectal carcinoma OR colorectal tumor OR colon cancer OR colon neoplasm OR rectal cancer OR rectum cancer OR rectal neoplasm OR	N/A	2021/4/25	310

	rectum neoplasm OR colon tumor OR rectum tumor OR rectal tumor) AND (random OR randomized OR randomised)			
ProQuest	(iron OR erythropoietin) AND (colorectal cancer) AND (random OR randomized OR randomised)	cancer/colorectal cancer	2021/4/25	584
ScienceDirect	(iron OR erythropoietin) AND (colorectal cancer) AND (random OR randomized OR randomised)	research article	2021/4/25	1893
Web of Science	(iron OR ferritin OR ferric OR ferrous OR erythropoietin OR epoetin alfa OR EPO OR haemopoietin OR haemopoietic OR epoetin beta OR haematopoietin OR erythrogenin OR erythrogenic OR erythropoetin) AND (colorectal cancer OR colorectal neoplasm OR colorectal carcinoma OR colorectal tumor OR colon cancer OR colon neoplasm OR rectal cancer OR rectum cancer OR rectal neoplasm OR rectum neoplasm OR colon tumor OR rectum tumor OR rectal tumor) AND (random OR randomized OR randomised)	N/A	2021/4/25	154
ClinicalTrials.gov	(iron OR erythropoietin) AND (colorectal cancer) AND (random OR randomized OR randomised)	N/A	2021/4/25	4

Abbreviation: N/A: not applied

Table S3: Excluded studies and reason

Reason	Numbers	References
Also included patients with other cancer but not only colorectal cancer	1	[1]
Commentary	2	[2,3]
Duplicate sample source with another included study	3	[4-6]
Intra- and post-operation intervention but not pre-operation intervention	1	[7]
Meta-analysis	2	[8,9]
Not associate with blood iron and hemoglobin related outcome	2	[10,11]
Not randomized controlled trials	3	[12-14]
Retrospective study but not randomized controlled trials	1	[15]
Review article	1	[16]
Study protocol but not result of a randomized controlled trial	4	[17-20]

References:

1. Glimelius, B.; Linne, T.; Hoffman, K.; Larsson, L.; Svensson, J.H.; Nasman, P.; Svensson, B.; Helmers, C. Epoetin beta in the treatment of anemia in patients with advanced gastrointestinal cancer. *J Clin Oncol* **1998**, *16*, 434-440, doi:10.1200/JCO.1998.16.2.434.
2. Ranganathan, P.; Pramesh, C.S. Letter 1: Randomized clinical trial of preoperative intravenous iron sucrose to reduce blood transfusion in anaemic patients after colorectal cancer surgery (Br J Surg 2009; 96: 1122-1128). *Br J Surg* **2010**, *97*, 297-298; author reply 299, doi:10.1002/bjs.6980.
3. Simpson, J.A.; Tselepis, C.; Iqbal, T.; Acheson, A.G.; Brookes, M.J. Letter 2: Randomized clinical trial of preoperative intravenous iron

sucrose to reduce blood transfusion in anaemic patients after colorectal cancer surgery (Br J Surg 2009; 96: 1122-1128). *Br J Surg* **2010**, *97*, 298-299; author reply 299, doi:10.1002/bjs.6981.
4. Dickson, E.A.; Keeler, B.D.; Ng, O.; Kumar, A.; Brookes, M.J.; Acheson, A.G.; group, I.t. Preoperative intravenous iron therapy and survival after colorectal cancer surgery: long-term results from the IVICA randomised controlled trial. *Colorectal Dis* **2020**, doi:10.1111/codi.15342.
5. Keeler, B.D.; Dickson, E.A.; Simpson, J.A.; Ng, O.; Padmanabhan, H.; Brookes, M.J.; Acheson, A.G.; Group, I.T. The impact of pre-operative intravenous iron on quality of life after colorectal cancer surgery: outcomes from the intravenous iron in colorectal cancer-associated anaemia (IVICA) trial. *Anaesthesia* **2019**, *74*, 714-725, doi:10.1111/anae.14659.
6. Qvist, N.; Boesby, S.; Wolff, B.; Hansen, C.P. [Perioperative administration of recombinant human erythropoietin in colorectal cancer surgery. A prospective, randomized, double-blind placebo controlled study]. *Ugeskr Laeger* **2000**, *162*, 355-358.
7. Kettelhack, C.; Hones, C.; Messinger, D.; Schlag, P.M. Randomized multicentre trial of the influence of recombinant human erythropoietin on intraoperative and postoperative transfusion need in anaemic patients undergoing right hemicolectomy for carcinoma. *Br J Surg* **1998**, *85*, 63-67, doi:10.1046/j.1365-2168.1998.00564.x.
8. Hallet, J.; Hanif, A.; Callum, J.; Pronina, I.; Wallace, D.; Yohanathan, L.; McLeod, R.; Coburn, N. The impact of perioperative iron on the use of red blood cell transfusions in gastrointestinal surgery: a systematic review and meta-analysis. *Transfus Med Rev* **2014**, *28*, 205-211, doi:10.1016/j.tmrv.2014.05.004.
9. Devon, K.M.; McLeod, R.S. Pre and peri-operative erythropoietin for reducing allogeneic blood transfusions in colorectal cancer surgery. *The Cochrane database of systematic reviews* **2009**, CD007148, doi:10.1002/14651858.CD007148.pub2.
10. Ferrari, P.; Nicolini, A.; Manca, M.L.; Rossi, G.; Anselmi, L.; Conte, M.; Carpi, A.; Bonino, F. Treatment of mild non-chemotherapy-induced iron deficiency anemia in cancer patients: comparison between oral ferrous bisglycinate chelate and ferrous sulfate. *Biomed Pharmacother* **2012**, *66*, 414-418, doi:10.1016/j.biopha.2012.06.003.
11. Kozu, T.; Iinuma, G.; Ohashi, Y.; Saito, Y.; Akasu, T.; Saito, D.; Alexander, D.B.; Iigo, M.; Kakizoe, T.; Tsuda, H. Effect of orally administered bovine lactoferrin on the growth of adenomatous colorectal polyps in a randomized, placebo-controlled clinical trial. *Cancer Prev Res*

(Phila) **2009**, *2*, 975-983, doi:10.1158/1940-6207.CAPR-08-0208.
12. Cornish, A.J.; Law, P.J.; Timofeeva, M.; Palin, K.; Farrington, S.M.; Palles, C.; Jenkins, M.A.; Casey, G.; Brenner, H.; Chang-Claude, J.; et al. Modifiable pathways for colorectal cancer: a mendelian randomisation analysis. *Lancet Gastroenterol Hepatol* **2020**, *5*, 55-62, doi:10.1016/S2468-1253(19)30294-8.
13. Wilson, M.J.; Dekker, J.W.T.; Buettner, S.; Harlaar, J.J.; Jeekel, J.; Schipperus, M.; Zwaginga, J.J. The effect of intravenous iron therapy on long-term survival in anaemic colorectal cancer patients: Results from a matched cohort study. *Surg Oncol* **2018**, *27*, 192-199, doi:10.1016/j.suronc.2018.03.005.
14. Okuyama, M.; Ikeda, K.; Shibata, T.; Tsukahara, Y.; Kitada, M.; Shimano, T. Preoperative iron supplementation and intraoperative transfusion during colorectal cancer surgery. *Surg Today* **2005**, *35*, 36-40, doi:10.1007/s00595-004-2888-0.
15. Titos-Arcos, J.C.; Soria-Aledo, V.; Carrillo-Alcaraz, A.; Ventura-Lopez, M.; Palacios-Munoz, S.; Pellicer-Franco, E. Is intravenous iron useful for reducing transfusions in surgically treated colorectal cancer patients? *World J Surg* **2012**, *36*, 1893-1897, doi:10.1007/s00268-012-1589-x.
16. Borstlap, W.A.; Stellingwerf, M.E.; Moolla, Z.; Musters, G.D.; Buskens, C.J.; Tanis, P.J.; Bemelman, W.A. Iron therapy for the treatment of preoperative anaemia in patients with colorectal carcinoma: a systematic review. *Colorectal Dis* **2015**, *17*, 1044-1054, doi:10.1111/codi.13110.
17. Laso-Morales, M.J.; Vives, R.; Vallejo-Tarrat, A.; Calo, N.; Valle-Beltran, A.; Pontes, C. Single dose of intravenous ferric carboxymaltose infusion versus multiple fractionated doses of intravenous iron sucrose in the treatment of postoperative anaemia in colorectal cancer patients: study protocol for a randomised controlled trial. *Trials* **2019**, *20*, 23, doi:10.1186/s13063-018-3125-2.
18. Michel, P.; Boige, V.; Andre, T.; Aparicio, T.; Bachet, J.B.; Dahan, L.; Guimbaud, R.; Lepage, C.; Manfredi, S.; Tougeron, D.; et al. Aspirin versus placebo in stage III or high-risk stage II colon cancer with PIK3CA mutation: A French randomised double-blind phase III trial (PRODIGE 50-ASPIK). *Digestive and liver disease : official journal of the Italian Society of Gastroenterology and the Italian Association for the Study of the Liver* **2018**, *50*, 305-307, doi:10.1016/j.dld.2017.12.023.
19. Borstlap, W.A.; Buskens, C.J.; Tytgat, K.M.; Tuynman, J.B.; Consten, E.C.; Tolboom, R.C.; Heuff, G.; van Geloven, A.A.; van Wagensveld,

B.A.; Wientjes, C.A.; et al. Erratum to: Multicentre randomized controlled trial comparing ferric(III) carboxymaltose infusion with oral iron supplementation in the treatment of preoperative anaemia in colorectal cancer patients. *BMC Surg* **2015**, *15*, 110, doi:10.1186/s12893-015-0090-5.
20. Borstlap, W.A.A.; Buskens, C.J.; Tytgat, K.; Tuynman, J.B.; Consten, E.C.J.; Tolboom, R.C.; Heuff, G.; van Geloven, N.; van Wagensveld, B.A.; CA, C.A.W.; et al. Multicentre randomized controlled trial comparing ferric(III)carboxymaltose infusion with oral iron supplementation in the treatment of preoperative anaemia in colorectal cancer patients. *BMC Surg* **2015**, *15*, 78, doi:10.1186/s12893-015-0065-6.

Table S4: characteristics of the included studies

Study	Comparison	Number	Mean age	Female (%)	Time of iron supplement	Baseline anemia	Duration of iron Tx	Country
Keeler, B.D. (2017)[1]	intravenous ferric carboxymaltose maximum 2000mg	55	73.8	36.4	Pre-op	Yes	21 days	UK
	oral iron 400mg	61	74.7	39.3				
Edwards, T.J. (2009)[2]	intravenous 600 mg iron sucrose	34	67.0	35.3	Pre-op	No	1 day	UK
	placebo (saline)	26	70.0	34.6				
Lidder, P.G. (2007)[3]	oral ferrous sulphate 200 mg TDS	24	69.0	33.3	Pre-op	No	14 days	UK
	standard clinical management	25	72.0	36.0				
Norager, C.B. (2006)[4]	darbepoetin alfa + Oral iron supplements (200mg/day)	75	65.0	44.0	Pre-, Peri-, or post-op*	Yes	total 35 days but 10 days before surgery	Denmark
	placebo + Oral iron supplements (200mg/day)	76	63.0	38.2				
Christodoulakis, M. (2005)[5]	epoetin alfa 150 IU/kg + Oral iron supplements (200mg/day)	69	72.5	55.1	Pre-, Peri-, or post-op*	Yes	total 12 days but 10 days before surgery	Greece
	epoetin alfa 300 IU/kg + Oral iron supplements (200mg/day)	67	71.0	55.2				
	Oral iron supplements (200mg/day)	68	70.0	58.8				
Qvist, N. (1999)[6]	recombinant human erythropoietin (epoetin alfa) 300 IU/kg + Oral iron supplements (200mg/day)	38	69.0	68.4	Pre-, Peri-, or post-op*	Yes	total 7 days but 4 days before surgery	Denmark
	placebo + Oral iron supplements (200mg/day)	43	69.0	53.5				
Heiss, M.M. (1996)[7]	human recombinant erythropoietin 150 IU/kg (Cilag GmbH product) + Oral ferrous sulfate supplements (200mg/day)	17	66.0	58.8	Pre-, Peri-, or post-op*	Yes	total 12 days but 10 days before surgery	Germany
	placebo + Oral ferrous sulfate supplements (200mg/day)	10	61.0	80.0				

*: we only extracted the outcome data just before the target surgery

References:

1. Keeler, B.D.; Simpson, J.A.; Ng, O.; Padmanabhan, H.; Brookes, M.J.; Acheson, A.G.; Group, I.T. Randomized clinical trial of preoperative oral versus intravenous iron in anaemic patients with colorectal cancer. *Br J Surg* **2017**, *104*, 214-221, doi:10.1002/bjs.10328.
2. Edwards, T.J.; Noble, E.J.; Durran, A.; Mellor, N.; Hosie, K.B. Randomized clinical trial of preoperative intravenous iron sucrose to reduce blood transfusion in anaemic patients after colorectal cancer surgery. *Br J Surg* **2009**, *96*, 1122-1128, doi:10.1002/bjs.6688.
3. Lidder, P.G.; Sanders, G.; Whitehead, E.; Douie, W.J.; Mellor, N.; Lewis, S.J.; Hosie, K.B. Pre-operative oral iron supplementation reduces blood transfusion in colorectal surgery - a prospective, randomised, controlled trial. *Ann R Coll Surg Engl* **2007**, *89*, 418-421, doi:10.1308/003588407X183364.
4. Norager, C.B.; Jensen, M.B.; Madsen, M.R.; Qvist, N.; Laurberg, S. Effect of darbepoetin alfa on physical function in patients undergoing surgery for colorectal cancer. A randomized, double-blind, placebo-controlled study. *Oncology* **2006**, *71*, 212-220, doi:10.1159/000106071.
5. Christodoulakis, M.; Tsiftsis, D.D.; Hellenic Surgical Oncology Perioperative, E.P.O.S.G. Preoperative epoetin alfa in colorectal surgery: a randomized, controlled study. *Ann Surg Oncol* **2005**, *12*, 718-725, doi:10.1245/ASO.2005.06.031.
6. Qvist, N.; Boesby, S.; Wolff, B.; Hansen, C.P. Recombinant human erythropoietin and hemoglobin concentration at operation and during the postoperative period: reduced need for blood transfusions in patients undergoing colorectal surgery--prospective double-blind placebo-controlled study. *World J Surg* **1999**, *23*, 30-35, doi:10.1007/s002689900561.
7. Heiss, M.M.; Tarabichi, A.; Delanoff, C.; Allgayer, H.; Jauch, K.W.; Hernandez-Richter, T.; Mempel, W.; Beck, K.G.; Schildberg, F.W.; Messmer, K. Perisurgical erythropoietin application in anemic patients with colorectal cancer: A double-blind randomized study. *Surgery* **1996**, *119*, 523-527, doi:10.1016/s0039-6060(96)80261-3.

Table S5A: SUCRA of the blood transfusion rate

Treatment	SUCRA
HighdoseEPO	95.4
LowdoseEPO	55.6
IViron	53.2
Oraliron	48.0
DarbEPO	40.5
Pla	7.2

Sorted by efficacy order (the former, the less blood transfusion rate)

Table S5B: SUCRA of the blood transfusion rate: subgroup of patients with baseline anemia

Treatment	SUCRA
HighdoseEPO	90.9
IViron	58.7
LowdoseEPO	41.1
Oraliron	31.3
DarbEPO	28.1

Sorted by efficacy order (the former, the less blood transfusion rate)

Table S5C: SUCRA of the improvement in hemoglobin

Treatment	SUCRA
HighdoseEPO	74.2
IViron	67.9
Oraliron	48.3
DarbEPO	40.5
LowdoseEPO	37.1
Pla	31.9

Sorted by efficacy order (the former, the better improvement in hemoglobin)

Table S5D: SUCRA of the improvement in ferritin

Treatment	SUCRA
IViron	80.6
Pla	51.5
Oraliron	17.9

Sorted by efficacy order (the former, the better improvement in ferritin)

Table S5E: SUCRA of the unit of blood transfused

Treatment	SUCRA
HighdoseEPO	2.4
LowdoseEPO	38.4
Oraliron	49.9
IViron	59.2
Pla	100.0

Sorted by efficacy order (the former, the less unit of blood transfused)

Table S5F: SUCRA of the drop out rate

Treatment	SUCRA
IViron	76.0
DarbEPO	69.4
Pla	56.1
Oraliron	50.4
HighdoseEPO	31.6
LowdoseEPO	16.5

Sorted by efficacy order (the former, the less drop out rate)

Abbreviation: AMSTAR: assessing the methodological quality of systematic review; CI: confidence interval; DarbEPO: darbepoetin alfa; GRADE: Grading of Recommendations Assessment, Development and Evaluation; HighdoseEPO: epoetin alfa 300 IU/kg; IViron: intravenous iron supplement; LowdoseEPO: epoetin alfa 150 IU/kg; MD: mean difference; NMA: network meta-analysis; OR: odds ratio; Oraliron: oral iron supplement; Pla: placebo/Control; PRISMA: preferred reporting items for systematic reviews and meta-analysis; RCT: randomized controlled trial; SUCRA: surface under the cumulative ranking curve

Table S6A: League table of the blood transfusion rate: subgroup of patients with baseline anemia

HighdoseEPO		0.61 (0.31,1.22)	***0.50 (0.29,0.86)**	
0.69 (0.24,1.99)	IViron		0.75 (0.30,1.85)	
0.56 (0.30,1.05)	0.81 (0.27,2.38)	LowdoseEPO	0.96 (0.52,1.77)	
***0.52 (0.30,0.89)**	0.75 (0.30,1.85)	0.93 (0.51,1.68)	Oraliron	0.87 (0.33,2.29)
0.45 (0.15,1.36)	0.65 (0.17,2.45)	0.81 (0.26,2.51)	0.87 (0.33,2.29)	DarbEPO

Pairwise (upper-right portion) and network (lower-left portion) meta-analysis results are presented as estimate effect sizes for the outcome of blood transfusion rate in patients with colorectal cancer. Interventions are reported in order of mean ranking of efficacy, and outcomes are expressed as odds ratio (OR) (95% confidence intervals). For the pairwise meta-analyses, OR of less than 1 indicate that the treatment specified in the row got better efficacy (i.e. less blood transfusion rate) than that specified in the column. For the network meta-analysis (NMA), OR of less than 1 indicate that the treatment specified in the column got better efficacy (i.e. less blood transfusion rate) than that specified in the row. Bold results marked with * indicate statistical significance.

Table S6B: League table of the improvement in hemoglobin

HighdoseEPO		***0.98 (0.35,1.62)**			
0.50 (-2.52,3.52)	IViron	1.12 (0.69,1.56)			0.31 (-0.43,1.05)
0.98 (-1.36,3.32)	0.48 (-1.44,2.39)	Oraliron	***0.31 (0.26,0.35)**	0.40 (-0.10,0.90)	***1.30 (0.18,2.42)**
1.29 (-1.96,4.53)	0.79 (-2.17,3.74)	0.31 (-1.94,2.56)	DarbEPO		
1.38 (-1.90,4.66)	0.88 (-2.12,3.87)	0.40 (-1.90,2.70)	0.09 (-3.12,3.31)	LowdoseEPO	
1.50 (-1.58,4.58)	1.00 (-0.94,2.94)	0.52 (-1.48,2.53)	0.21 (-2.80,3.23)	0.12 (-2.93,3.18)	Pla

Pairwise (upper-right portion) and network (lower-left portion) meta-analysis results are presented as estimate effect sizes for the outcome of improvement in hemoglobin in patients with colorectal cancer. Interventions are reported in order of mean ranking of hemoglobin improvement, and outcomes are expressed as mean difference (MD) (95% confidence intervals). For the pairwise meta-analyses, MD of higher than 0 indicate that the treatment specified in the row got more improvement than that specified in the column. For the network meta-analysis (NMA), MD of higher than 0 indicate that the treatment specified in the column got more improvement than that specified in the row. Bold results marked with * indicate statistical significance.

Table S6C: League table of the improvement in ferritin

IViron	-36.00 (-165.49,93.49)	***631.44 (479.40,783.48)**
194.82 (-443.03,832.68)	Pla	-22.24 (-32.85,77.32)
398.22 (-241.32,1037.77)	203.40 (-430.91,837.71)	Oraliron

Pairwise (upper-right portion) and network (lower-left portion) meta-analysis results are presented as estimate effect sizes for the outcome of improvement in ferritin in patients with colorectal cancer. Interventions are reported in order of mean ranking of ferritin improvement, and outcomes are expressed as mean difference (MD) (95% confidence intervals). For the pairwise meta-analyses, MD of higher than 0 indicate that the treatment specified in the row got more improvement than that specified in the column. For the network meta-analysis (NMA), MD of higher than 0 indicate that the treatment specified in the column got more improvement than that specified in the row. Bold results marked with * indicate statistical significance.

Table S6D: League table of the improvement in unit of blood transfused

HighdoseEPO	-0.380 (-0.83,0.07)	***-0.53 (-1.01,-0.05)**		
-0.41 (-0.84,0.02)	LowdoseEPO	-0.09 (-0.51,0.32)		
***-0.50 (-0.95,-0.05)**	-0.09 (-0.51,0.32)	Oraliron	-0.07 (-0.73,0.60)	***-2.38 (-3.56,-1.21)**
-0.65 (-1.40,0.11)	-0.24 (-0.97,0.49)	-0.15 (-0.75,0.46)	IViron	***-1.82 (-2.74,-0.91)**
***-2.62 (-3.55,-1.70)**	***-2.22 (-3.13,-1.31)**	***-2.13 (-2.94,-1.31)**	***-1.98 (-2.73,-1.22)**	Pla

Pairwise (upper-right portion) and network (lower-left portion) meta-analysis results are presented as estimate effect sizes for the outcome of improvement in unit of blood transfused in patients with colorectal cancer. Interventions are reported in order of mean ranking of improvement in unit of blood transfused, and outcomes are expressed as mean difference (MD) (95% confidence intervals). For the pairwise meta-analyses, MD of less than 0 indicate that the treatment specified in the row got more improvement than that specified in the column. For the network meta-analysis (NMA), MD of less than 0 indicate that the treatment specified in the column got more improvement than that specified in the row. Bold results marked with * indicate statistical significance.

Table S6E: League table of the drop out rate

IViron		0.77 (0.05,12.81)	0.54 (0.10,3.06)		
0.74 (0.14,4.00)	DarbEPO		0.77 (0.41,1.46)		
0.65 (0.09,4.55)	0.87 (0.14,5.59)	Pla	0.96 (0.12,7.41)		
0.57 (0.12,2.71)	0.77 (0.41,1.46)	0.88 (0.15,5.01)	Oraliron	0.64 (0.23,1.76)	0.20 (0.01,4.24)
0.37 (0.06,2.35)	0.49 (0.15,1.63)	0.56 (0.08,4.22)	0.64 (0.23,1.76)	HighdoseEPO	
0.11 (0.00,3.52)	0.15 (0.01,3.48)	0.17 (0.01,5.90)	0.20 (0.01,4.24)	0.31 (0.01,7.76)	LowdoseEPO

Pairwise (upper-right portion) and network (lower-left portion) meta-analysis results are presented as estimate effect sizes for the outcome of drop out rate in patients with colorectal cancer. Interventions are reported in order of mean ranking of acceptability, and outcomes are expressed as odds ratio (OR) (95% confidence intervals). For the pairwise meta-analyses, OR of less than 1 indicate that the treatment specified in the row got better acceptability (i.e. less drop out rate) than that specified in the column. For the network meta-analysis (NMA), OR of less than 1 indicate that the treatment specified in the column got better acceptability (i.e. less drop out rate) than that specified in the row. Bold results marked with * indicate statistical significance.

Abbreviation: AMSTAR: assessing the methodological quality of systematic review; CI: confidence interval; DarbEPO: darbepoetin alfa; GRADE: Grading of Recommendations Assessment, Development and Evaluation; HighdoseEPO: epoetin alfa 300 IU/kg; IViron: intravenous iron supplement; LowdoseEPO: epoetin alfa 150 IU/kg; MD: mean difference; NMA: network meta-analysis; OR: odds ratio; Oraliron: oral iron supplement; Pla: placebo/Control; PRISMA: preferred reporting items for systematic reviews and meta-analysis; RCT: randomized controlled trial; SUCRA: surface under the cumulative ranking curve

Table S7: Inconsistency of different intervention

Part of rate of blood transfusion

Side	nosymmetric		symmetric		Treatments used	
	P>z	tau	P>z	tau		
A B	0.269	1.03E-07	0.269	1.03E-07	A (reference):	Pla
A C	0.269	2.13E-07	0.269	2.13E-07	B:	Oraliron
B C	0.269	1.86E-08	0.269	1.42E-07	C:	IViron
B D	.	.	0.423	1.06E-07	D:	HighdoseEPO
B E	.	.	0.716	1.36E-05	E:	LowdoseEPO
B F	.	.	0.999	3.01E-05	F:	DarbEPO
D E	0.423	6.66E-06	0.48	2.07E-07		

Part of changes of hemoglobin

Side	nosymmetric		symmetric		Treatments used	
	P>z	tau	P>z	tau		
A B	0.003	4.33E-05	0.003	4.33E-05	A (reference):	Pla
A C	0.003	0.000164	0.003	0.000164	B:	Oraliron
B C	0.003	4.46E-05	0.003	0.000214	C:	IViron
B D	.	.	0.998	1.147073	D:	HighdoseEPO

B E	.	.	0.999	1.147125	E:	LowdoseEPO
B F	.	.	0.993	1.147956	F:	DarbEPO

Part of changes of ferritin

Side	nosymmetric		symmetric		Treatments used	
	P>z	tau	P>z	tau		
A B	0	0.445877	0	0.445877	A (reference):	Pla
A C	0	0.442876	0	0.442876	B:	Oraliron
B C	0	0.264258	0	0.121091	C:	IViron

Part of changes of amounts of blood transfused

Side	nosymmetric		symmetric		Treatments used	
	P>z	tau	P>z	tau		
A B	0.551	1.42E-06	0.551	1.42E-06	A (reference):	Pla
A C	0.551	1.41E-06	0.551	1.41E-06	B:	Oraliron
B C	0.551	3.97E-06	0.551	4.01E-07	C:	IViron
B D	.	.	0.704	1.54E-07	D:	HighdoseEPO
B E	.	.	0.992	3.15E-07	E:	LowdoseEPO
D E *	0.704	2.51E-05	0.704	6.60E-07		

Part of acceptability (i.e. drop out rate)

Side	nosymmetric		symmetric		Treatments used	
	P>z	tau	P>z	tau		
A B	0.877	0.002621	0.877	0.002621	A (reference):	Pla
A C	0.877	0.000363	0.877	0.000363	B:	Oraliron
B C	0.877	0.002707	0.877	0.000572	C:	IViron
B D	.	.	1	8.05E-06	D:	HighdoseEPO
B E	.	.	0.999	4.24E-07	E:	LowdoseEPO
B F	.	.	1	1.23E-06	F:	DarbEPO

Abbreviation: AMSTAR: assessing the methodological quality of systematic review; CI: confidence interval; DarbEPO: darbepoetin alfa; GRADE: Grading of Recommendations Assessment, Development and Evaluation; HighdoseEPO: epoetin alfa 300 IU/kg; IViron: intravenous iron supplement; LowdoseEPO: epoetin alfa 150 IU/kg; MD: mean difference; NMA: network meta-analysis; OR: odds ratio; Oraliron: oral iron supplement; Pla: placebo/Control; PRISMA: preferred reporting items for systematic reviews and meta-analysis; RCT: randomized controlled trial; SUCRA: surface under the cumulative ranking curve

Table S8: Estimated between-studies standard deviation of different outcome

Outcome	Estimated between-studies standard deviation
Rate of blood transfusion	1.086e-07
Changes of hemoglobin	1.1470647
Changes of ferritin	393.47632
Changes of amounts of blood transfused	2.528e-08
Acceptability (i.e. drop out rate)	2.346e-07

Table S9: Quality of evidence for primary outcome: rate of blood transfusion

We evaluated the quality of evidence followed the articles of GRADE Working Group and of Cipriani, A [1,2].

Comparisons (study number)	GRADE Direct OR (95%CIs)	The final rating of direct evidence	Indirect Co-efficient (SE)	The final rating of indirect evidence	Network meta-analysis OR (95%CIs)	Overall quality of evidence
HighdoseEPO vs LowdoseEPO	0.61 (0.31,1.22)	⊕⊕◯◯ low	1.13 (0.84)	⊕⊕◯◯ low	0.56 (0.30,1.05)	⊕⊕⊕◯ medium
HighdoseEPO vs IViron					0.55 (0.21,1.46)	⊕◯◯◯ very low
HighdoseEPO vs Oraliron	***0.50 (0.29,0.86)**	⊕⊕⊕◯ medium	0.70 (1.72)	⊕◯◯◯ very low	***0.52 (0.30,0.89)**	⊕⊕⊕⊕ high
HighdoseEPO vs DarbEPO					0.45 (0.15,1.36)	⊕◯◯◯ very low
HighdoseEPO vs Pla					***0.24 (0.08,0.73)**	⊕⊕◯◯ low
LowdoseEPO vs IViron					0.99 (0.36,2.70)	⊕◯◯◯ very low
LowdoseEPO vs Oraliron	0.96 (0.52,1.77)	⊕◯◯◯ very low	-0.46 (1.10)	⊕⊕◯◯ low	0.93 (0.51,1.68)	⊕⊕⊕◯ medium
LowdoseEPO vs DarbEPO					0.81 (0.26,2.51)	⊕◯◯◯ very low
LowdoseEPO vs Pla					0.43 (0.14,1.34)	⊕◯◯◯ very low
IViron vs Oraliron	0.75 (0.30,1.85)	⊕◯◯◯ very low	0.86 (0.93)	⊕⊕◯◯ low	0.94 (0.42,2.11)	⊕⊕⊕◯ medium
IViron vs DarbEPO					0.82 (0.23,2.89)	⊕◯◯◯ very low
IViron vs Pla	0.72 (0.19,2.82)	⊕◯◯◯ very low	-1.47 (0.77)	⊕⊕◯◯ low	0.43 (0.16,1.19)	⊕⊕⊕◯ medium
Oraliron vs DarbEPO	0.87 (0.33,2.29)	⊕◯◯◯ very low	1.65 (1742.10)	⊕◯◯◯ very low	0.87 (0.33,2.29)	⊕⊕⊕◯ medium

Oraliron vs Pla	0.31 (0.09,1.03)	⊕⊕◯◯ low	-0.03 (0.83)	⊕⊕◯◯ low	0.46 (0.17,1.22)	⊕⊕⊕◯ medium
DarbEPO vs Pla					0.53 (0.13,2.08)	⊕◯◯◯ very low

Abbreviation: AMSTAR: assessing the methodological quality of systematic review; CI: confidence interval; DarbEPO: darbepoetin alfa; GRADE: Grading of Recommendations Assessment, Development and Evaluation; HighdoseEPO: epoetin alfa 300 IU/kg; IViron: intravenous iron supplement; LowdoseEPO: epoetin alfa 150 IU/kg; MD: mean difference; NMA: network meta-analysis; OR: odds ratio; Oraliron: oral iron supplement; Pla: placebo/Control; PRISMA: preferred reporting items for systematic reviews and meta-analysis; RCT: randomized controlled trial; SE: standard error; SUCRA: surface under the cumulative ranking curve

Reference:

1. Cipriani, A.; Furukawa, T.A.; Salanti, G.; Chaimani, A.; Atkinson, L.Z.; Ogawa, Y.; Leucht, S.; Ruhe, H.G.; Turner, E.H.; Higgins, J.P.T.; et al. Comparative efficacy and acceptability of 21 antidepressant drugs for the acute treatment of adults with major depressive disorder: a systematic review and network meta-analysis. *Lancet* **2018**, doi:10.1016/S0140-6736(17)32802-7.
2. Puhan, M.A.; Schunemann, H.J.; Murad, M.H.; Li, T.; Brignardello-Petersen, R.; Singh, J.A.; Kessels, A.G.; Guyatt, G.H.; Group, G.W. A GRADE Working Group approach for rating the quality of treatment effect estimates from network meta-analysis. *Bmj* **2014**, *349*, g5630, doi:10.1136/bmj.g5630.
